# Supplementary material for: WaveSeekerNet: accurate prediction of influenza A virus subtypes and host source using attention-based deep learning
Source: Gigascience. 2025 Aug 29;14:giaf089. doi: 10.1093/gigascience/giaf089 (PMC12395966; doi:10.1093/gigascience/giaf089)
Supplement: giaf089_Supplemental_Files [file giaf089_supplemental_files.zip › Supplementary Algorithms_Rev1.pdf]

Supplementary Materials

Algorithm S1: An algorithm to transform a DNA/RNA sequence into a chaos game representation.

Input:<br>S - A sequence such as that $S = s_1, s_2, ..., s_i \in \{A, C, G, T/U\}$<br>$P_{i-1}$ - The *x* and *y* coordinates of the nucleotide found at position *i-1* in sequence *S*.
For s_i in S:<br>If $i = 1$:<br>$P_i = 0.5[(0, 0) + g(s_i)]$<br>Else:<br>$P_i = 0.5[P_{i-1} + g(s_i)]$<br>Where:<br>$g(s)\ is\ (1, 1)\ if\ s = A,\ (-1, 1)\ if\ s = C,\ (-1, -1)\ if\ s = G,\ or\ (1, -1)\ if\ s = T/U$
Output:<br>A chaos game representation of *S*

Algorithm S2: Pseudo-code for the Fourier Transform block.

```
def FFTBlock(x):
    x = RFFT2(x)

    x = proj(x, d)
    x = reshape(b*h, n, 32)
    x = x + attn_dropout(efficient_attention(x), 0.125)
    x = RMSNorm(x)
    x = NoisyFactoriedLinear(x, 32) + x
    x = reshape(b, n, d)
    x = merge(b, n, d//2+1)
    x = shrinkage(x)

    x = IRFFT2(x)
    x = scale(x, -5, 5)
    x = FFN(x)
    x = Dropout1d(0.125)

    return x
```

Supplementary Materials

Algorithm S3: Pseudo-code for the Wavelet Transform block.

```
def DWTBlock(x):

    x = init_proj(x, d)
    x = reshape(b, h, n, 32)
    y_L, y_H = DWT(x)

    x_L = StartNet(y_L) + y_L
    x_L = RMSNorm(x_L)
    x_L = FFN(x_L) + x_L

    x_H = StarNet(y_H) + y_H
    x_H = RMSNorm(x_H)
    x_H = Linear(x_H) + x_H
    x_H = shrinkage(x_H)

    x = iDWT(x_L, x_H)
    x = reshape(b, n, d)
    x = FFN(x)
    x = Dropout1d(0.125)

    return x
```

Algorithm S4: Calculation of the Router Z-loss.

To ensure that experts are utilized efficiently, we use the router z-loss (Equation 1), which penalizes large logits before the router in the ST-MoE (Stable and Transferable Mixture-of-Experts) network. This helps increase the utilization of experts at each layer [1]. In this equation, *x* represents the logits, *T* is the number of tokens, and *N* is the number of experts. A scaling $\frac{1}{10}$ is used so that this loss does not overwhelm the cross-entropy loss.

$$Z\ Loss\ (x) = \frac{\frac{1}{T}\sum_{i=1}^{T}\left(log \sum_{j=1}^{N} e^{x_j^{(i)}}\right)^2}{10} \quad (1)$$

Algorithm S5: Calculation of the KAN Regularization Loss.

L1 regularization can be used to favor sparsity and simplicity within feed-forward networks. This helps prevent overfitting and can generally improve the robustness of models. Within the KAN, linear weights are replaced with learnable activation functions [2], and regularization forces the network to select the most appropriate activation functions.

Supplementary Materials

The first part of the KAN loss involves calculating the L1-norms of all the activation functions [2]. The L1-norm of the spline weights vector, $|W|_1$, can be approximated by taking the absolute value of the spline weights tensor, W, and then dividing this by the size of the last dimension of the spline weight tensor, d_{spline} (Equation 2).

$$|W|_1 = \sum_{i=1}^{d_{in}} \sum_{j=1}^{d_{out}} \frac{\sum_{k=1}^{d_{spline}} \left|W_{i,j,k}\right|}{d_{spline}} \quad (2)$$

To induce sparsity, L1 regularization is not sufficient on its own and entropy regularization is also required [2]. This can be calculated according to Equation 3.

$$S(W) = -\sum_{i=1}^{d_{in}} \sum_{j=1}^{d_{out}} \frac{\frac{\sum_{k=1}^{d_{spline}} \left|W_{i,j,k}\right|}{d_{spline}}}{|W|_1} log\left(\frac{\frac{\sum_{k=1}^{d_{spline}} \left|W_{i,j,k}\right|}{d_{spline}}}{|W|_1}\right) \quad (3)$$

The final KAN loss is calculated according to Equation 4, where L is the number of KAN layers in the classification head. A scaling $\frac{1}{100}$ is used so that this loss does not overwhelm the cross-entropy loss.

$$KAN\ Loss = \frac{\sum_{i=1}^{L} \left(\left|W_i\right|_1 + S(W_i)\right)}{100L} \quad (4)$$

References

[1] Zoph B, Bello I, Kumar S, Du N, Huang Y, Dean J, et al. ST-MoE: Designing Stable and Transferable Sparse Expert Models 2022. https://doi.org/10.48550/arXiv.2202.08906.

[2] Liu Z, Wang Y, Vaidya S, Ruehle F, Halverson J, Soljačić M, et al. KAN: Kolmogorov-Arnold Networks 2024. https://doi.org/10.48550/arXiv.2404.19756.
