# Supplementary material for: WaveSeekerNet: accurate prediction of influenza A virus subtypes and host source using attention-based deep learning
Source: Gigascience. 2025 Aug 29;14:giaf089. doi: 10.1093/gigascience/giaf089 (PMC12395966; doi:10.1093/gigascience/giaf089)

Supplementary Materials

Figure S1: The generalization performance of WaveSeekerNet for host source prediction was evaluated on the HA segment using various hyperparameter settings. The Balanced Accuracy, F1-score (Macro Average), and MCC are reported for high-quality (a) and low-quality (b) FCGR representations of RNA sequences. Scores for tests on the datasets constructed from high-quality and low-quality one-hot encoded protein sequences are reported in panels (c) and (d), respectively.

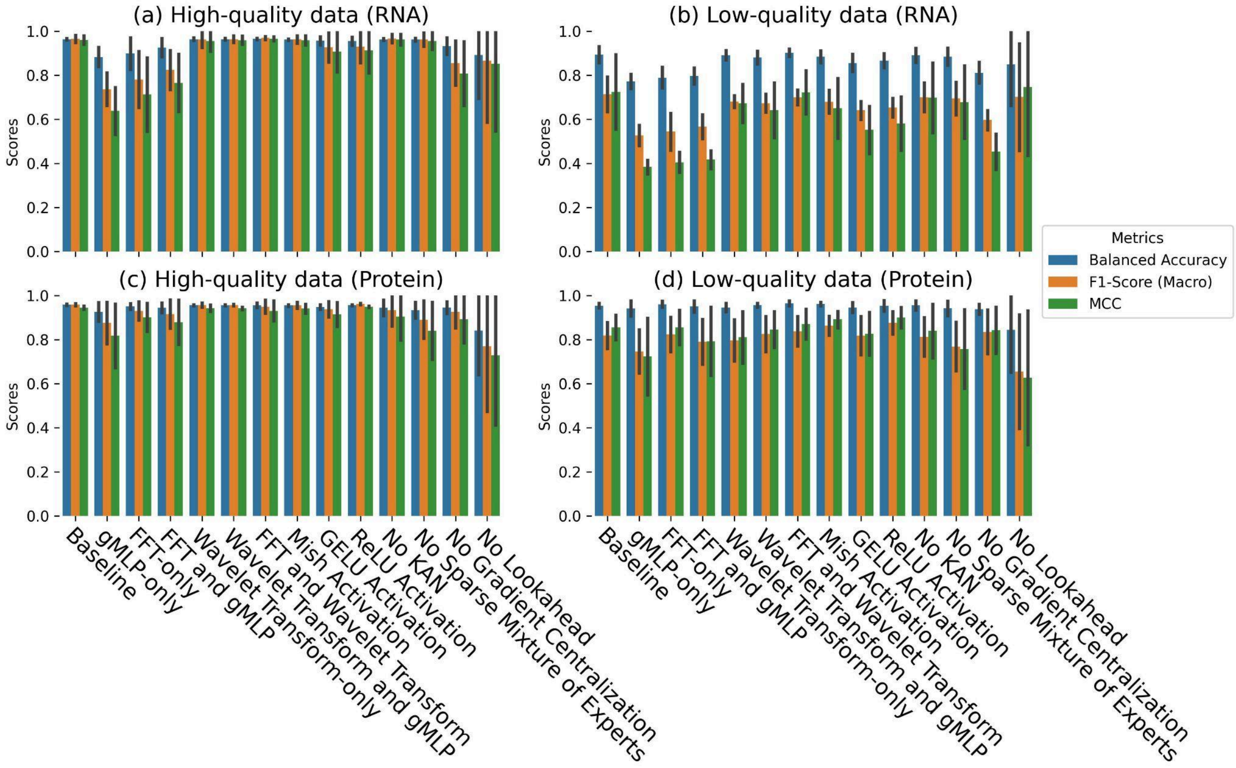

Supplementary Materials

Figure S2: The generalization performance of WaveSeekerNet for host source prediction was evaluated on the NA segment using various hyperparameter settings. The Balanced Accuracy, F1-score (Macro Average), and MCC are reported for high-quality (a) and low-quality (b) FCGR representations of RNA sequences. Scores for tests on the datasets constructed from high-quality and low-quality one-hot encoded protein sequences are reported in panels (c) and (d), respectively.

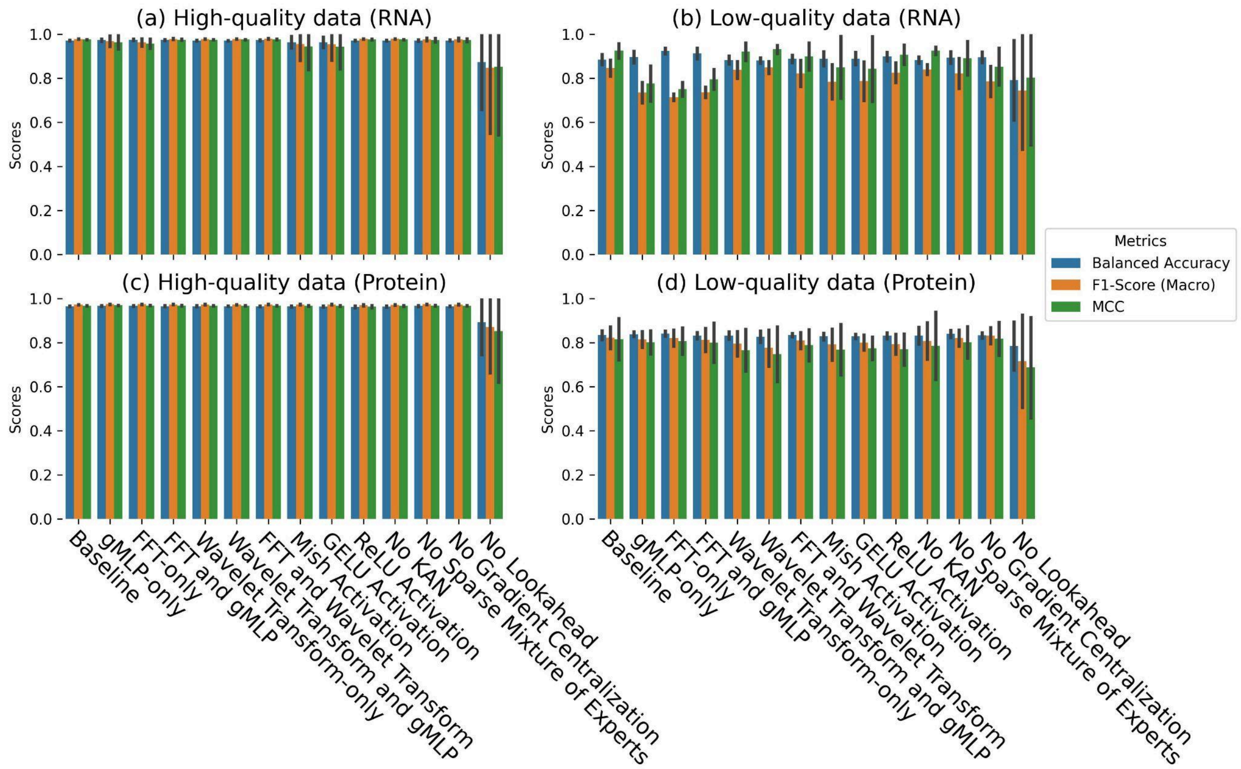

Supplementary Materials

Figure S3: The generalization performance of WaveSeekerNet for host source prediction was evaluated on the combined HA and NA segments (2 channels) using various hyperparameter settings. The Balanced Accuracy, F1-score (Macro Average), and MCC are reported for high-quality (a) and low-quality (b) FCGR representations of RNA sequences. Scores for tests on the datasets constructed from high-quality and low-quality one-hot encoded protein sequences are reported in panels (c) and (d), respectively.

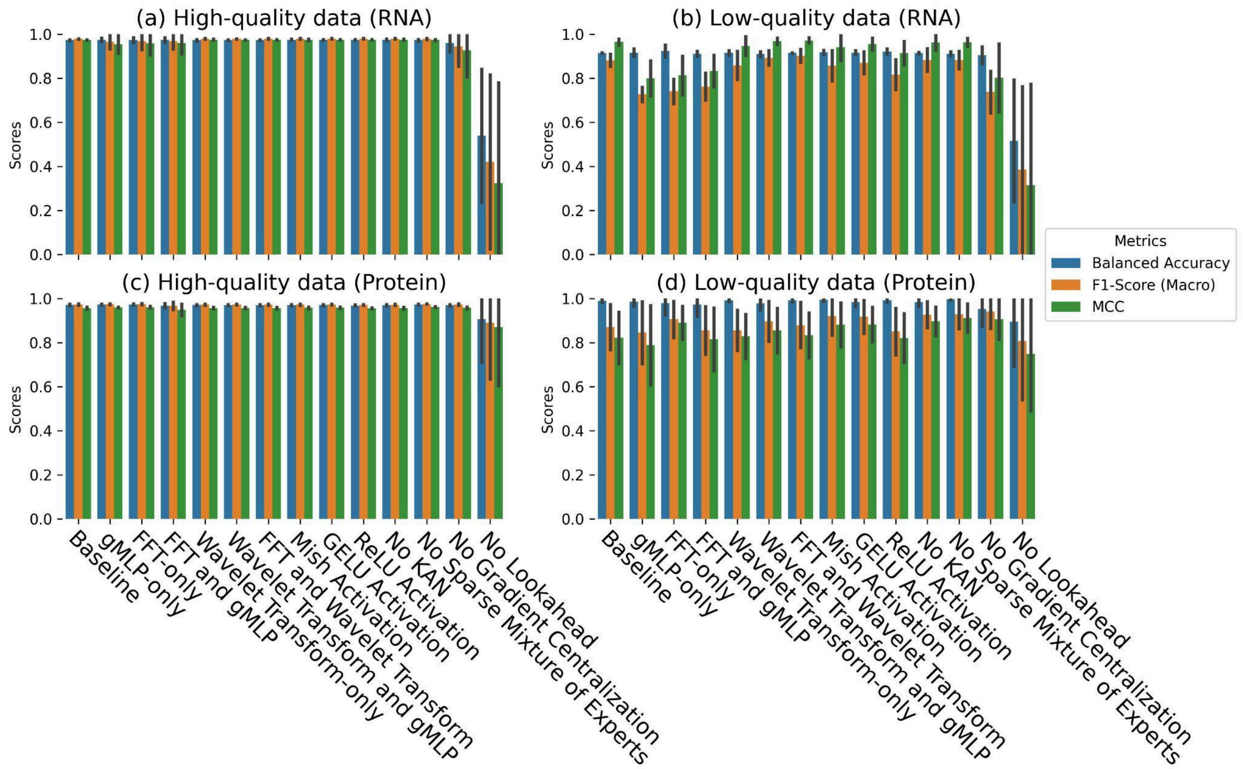

Supplementary Materials

Figure S4: The generalization performance of WaveSeekerNet and Transformer-only for HA subtype prediction using various hyperparameter settings. The Balanced Accuracy, F1-score (Macro Average), and MCC are reported for high-quality (a) and low-quality (b) FCGR representations of RNA sequences. Panels (c) and (d) show scores for high-quality and low-quality one-hot encoded protein sequences, respectively. The WaveSeekerNet and Transformer-only models using various hyperparameter settings are labeled in Black and Blue, respectively. The F1-scores (Macro Average) for VADR and BLASTp are shown as red horizontal dashed lines.

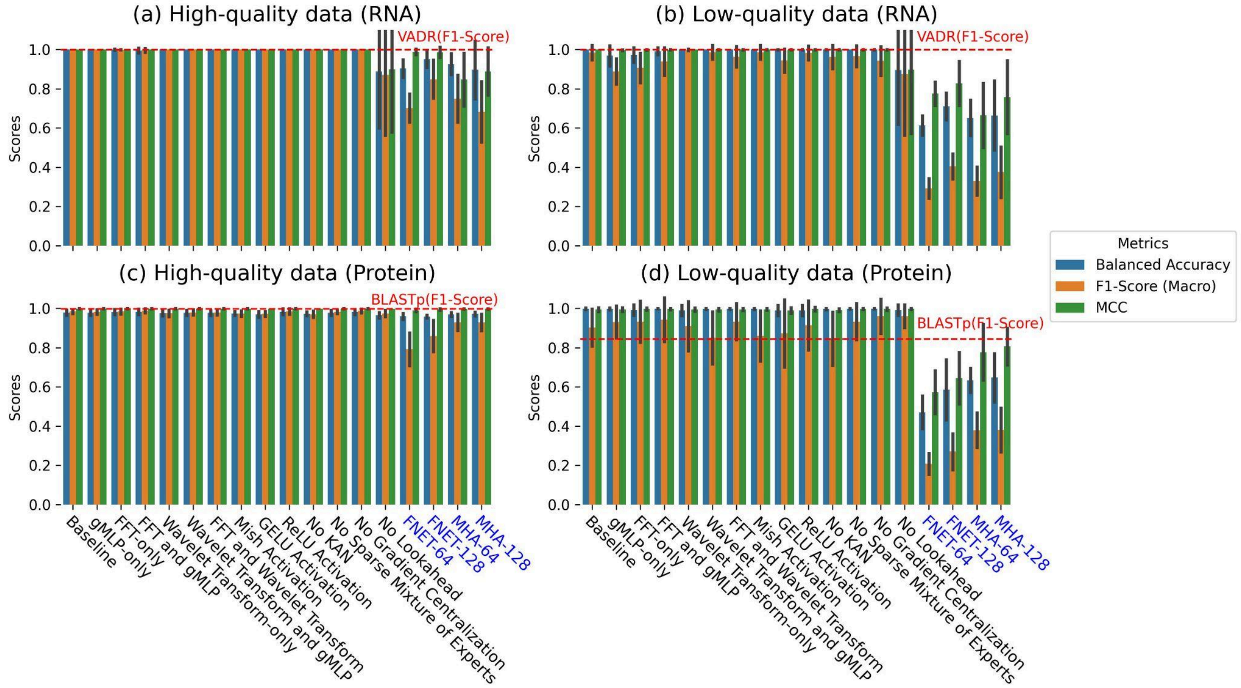

Supplementary Materials

Figure S5: The generalization performance of WaveSeekerNet and Transformer-only for NA subtype prediction using various hyperparameter settings. The Balanced Accuracy, F1-score (Macro Average), and MCC are reported for high-quality (a) and low-quality (b) FCGR representations of RNA sequences. Panels (c) and (d) show scores for high-quality and low-quality one-hot encoded protein sequences, respectively. The WaveSeekerNet and Transformer-only models using various hyperparameter settings are labeled in Black and Blue, respectively. The F1-scores (Macro Average) for VADR and BLASTp are shown as red horizontal dashed lines.

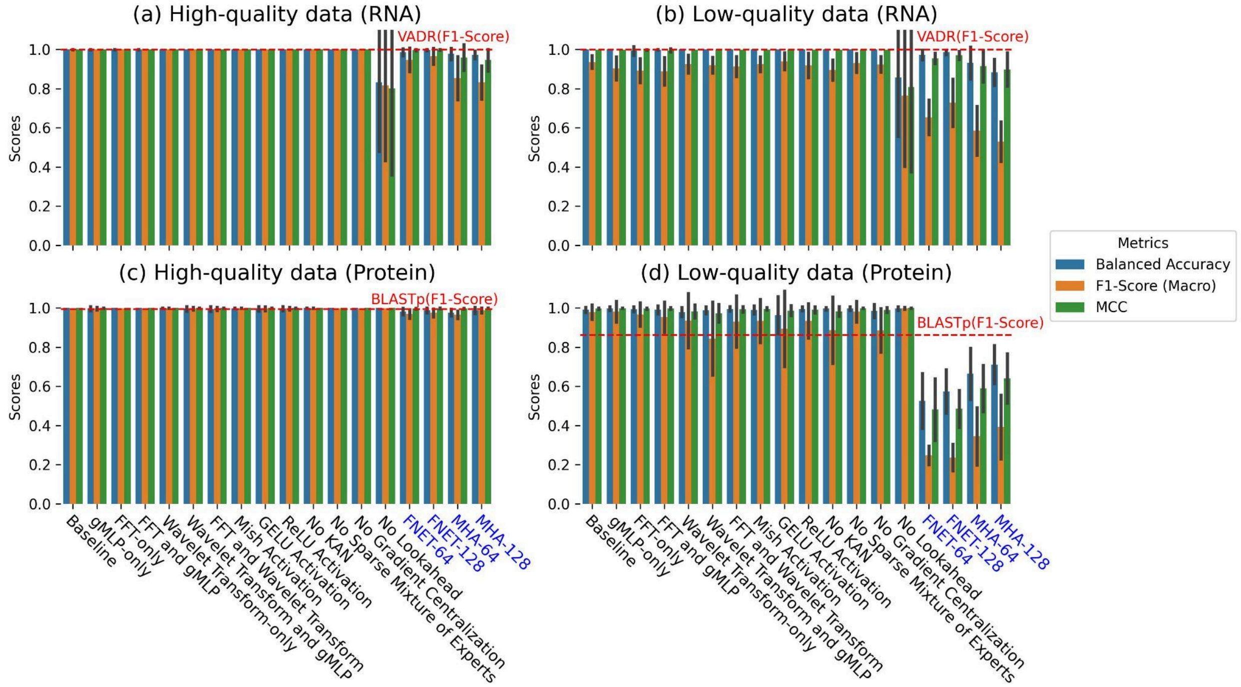

Supplementary Materials

Figure S6: Performance comparison of NA subtype prediction when the best-performing WaveSeekerNet, pre-trained ESM-2, and Transformer-only models are tested. The performance of the baseline WaveSeekerNet is also shown as a point of reference. The bar plots of Balanced Accuracy, F1-score (Macro Average), and MCC are reported for the high-quality (a) and low-quality (b) FCGR representations of RNA sequences. The bar plots of scores for tests on the datasets constructed from high-quality and low-quality one-hot encoded protein sequences are reported in panels (c) and (d), respectively. Horizontal dashed lines present the F1-scores (Macro Average) for VADR and BLASTp. The WaveSeekerNet, pre-trained ESM-2, and Transformer-only models are labeled in Black, Orange, and Blue, respectively.

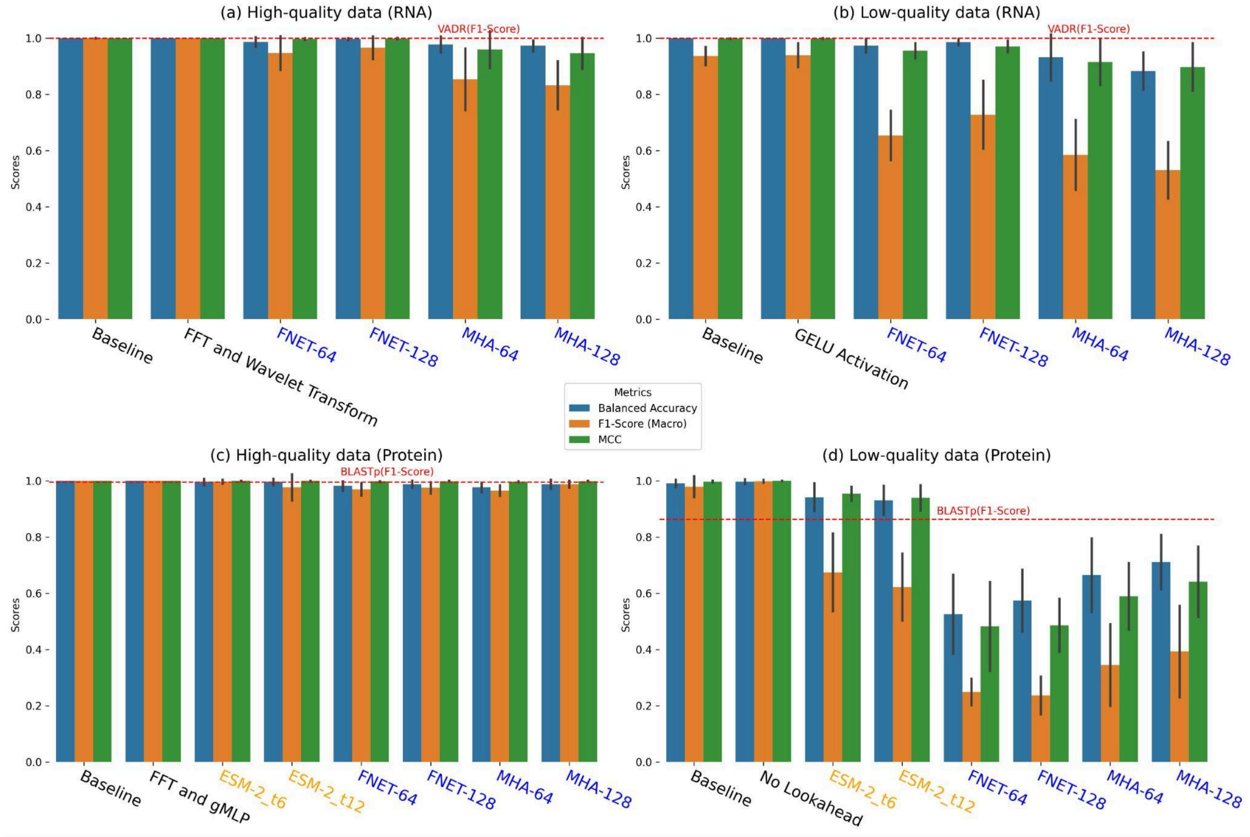

Supplementary Materials

Figure S7: Performance comparison of host source prediction using the NA segment when the best-performing WaveSeekerNet, pre-trained ESM-2, and Transformer-only models are tested. The performance of the baseline WaveSeekerNet is also shown as a point of reference. The bar plots of Balanced Accuracy, F1-score (Macro Average), and MCC are reported for the high-quality (a) and low-quality (b) FCGR representations of RNA sequences. The bar plots of scores for tests on the datasets constructed from high-quality and low-quality one-hot encoded protein sequences are reported in panels (c) and (d), respectively. The WaveSeekerNet, pre-trained ESM-2, and Transformer-only models are labeled in Black, Orange, and Blue, respectively.

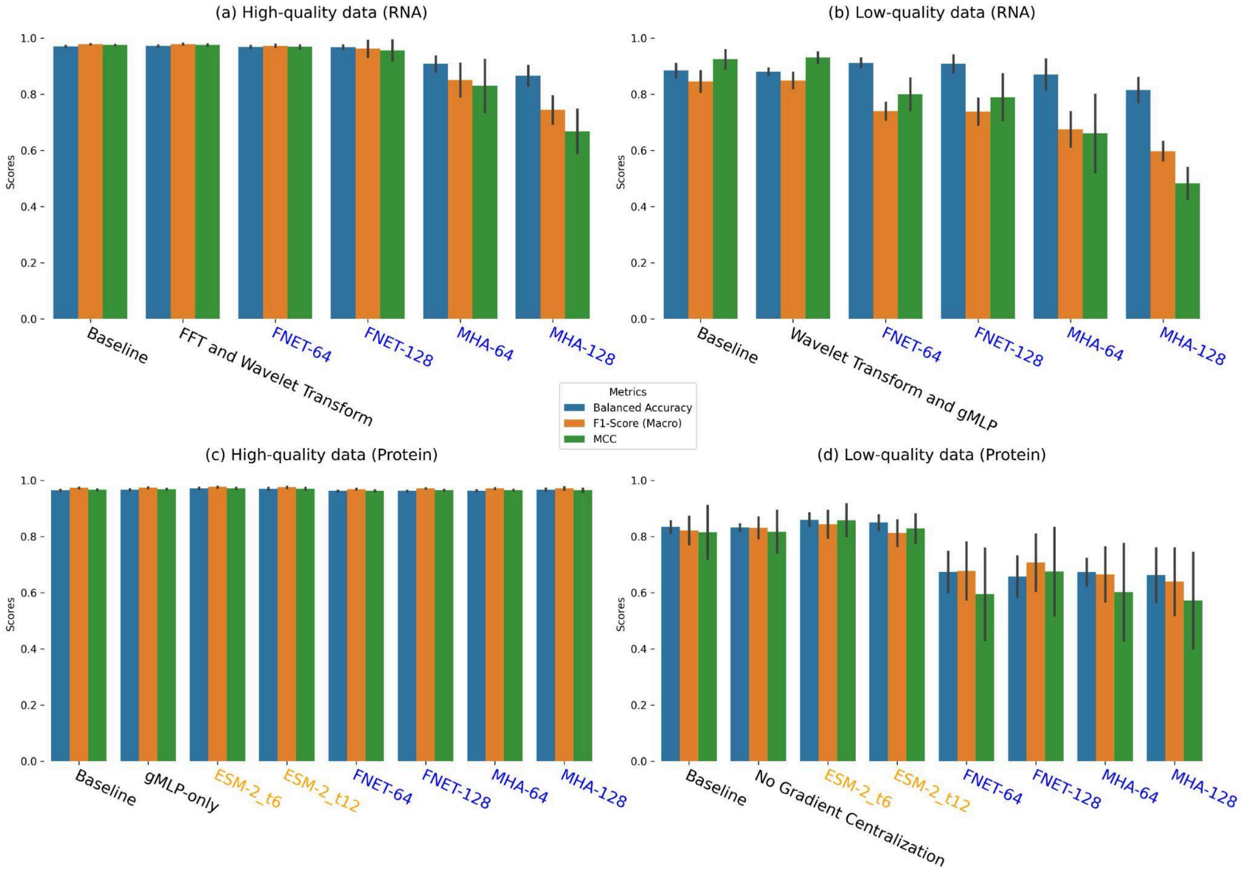

Supplement: giaf089_Supplemental_Files [file giaf089_supplemental_files.zip › Supplementary Figures_Rev1.pdf]
