# Supplementary material for: WaveSeekerNet: accurate prediction of influenza A virus subtypes and host source using attention-based deep learning
Source: Gigascience. 2025 Aug 29;14:giaf089. doi: 10.1093/gigascience/giaf089 (PMC12395966; doi:10.1093/gigascience/giaf089)
Supplement: giaf089_GIGA-D-25-00080_original_submission [file giaf089_giga-d-25-00080_original_submission.pdf]

GigaScience

WaveSeekerNet: Accurate Prediction of Influenza A Virus Subtypes and Host Source Using Attention-Based Deep Learning

--Manuscript Draft--

**Manuscript Number:**	GIGA-D-25-00080
**Full Title:**	WaveSeekerNet: Accurate Prediction of Influenza A Virus Subtypes and Host Source Using Attention-Based Deep Learning
**Article Type:**	Technical Note
**Funding Information:**	
**Abstract:**	Background<br>Influenza A virus (IAV) poses a significant threat to animal health globally, with its ability to overcome species barriers and cause pandemics. Rapid and accurate prediction of IAV subtypes and host source is crucial for effective surveillance and pandemic preparedness. Deep learning has emerged as a powerful tool for analyzing viral genomic sequences, offering new ways to uncover hidden patterns associated with viral characteristics and host adaptation.<br>Findings<br>We introduce WaveSeekerNet, a novel deep learning model for accurate and rapid prediction of IAV subtypes and host source. The model leverages attention-based mechanisms and efficient token mixing schemes, including the Fast Fourier Transform and the Wavelet Transform, to capture intricate patterns within viral RNA and protein sequences. Extensive experiments on diverse datasets demonstrate WaveSeekerNet's superior performance compared to existing Transformer-only models. Notably, WaveSeekerNet achieves scores of up to the maximum 1.0 across all evaluation metrics, including F1-score (Macro Average), Balanced Accuracy and Matthews Correlation Coefficient (MCC), in subtype prediction, even for rare subtypes. Furthermore, WaveSeekerNet exhibits remarkable accuracy in distinguishing between human, avian, and other mammalian hosts. The ability of WaveSeekerNet to flag potential cross-species transmission events underscores its significant value for real-time surveillance and proactive pandemic preparedness efforts.<br>Conclusions<br>WaveSeekerNet's superior performance, efficiency, and ability to flag potential cross-species transmission events highlight its potential for real-time surveillance and pandemic preparedness. This model represents a significant advancement in applying deep learning for IAV classification and holds promise for future epidemiological and veterinary studies, and public health interventions.
**Corresponding Author:**	Hoang-Hai Nguyen<br>University of Manitoba Faculty of Science<br>Winnipeg, Manitoba CANADA
**Corresponding Author Secondary Information:**	
**Corresponding Author's Institution:**	University of Manitoba Faculty of Science
**Corresponding Author's Secondary Institution:**	
**First Author:**	Hoang-Hai Nguyen
**First Author Secondary Information:**	
**Order of Authors:**	Hoang-Hai Nguyen
	Josip Rudar
	Nathaniel Lesperance
	Oksana Vernygora
	Graham W. Taylor
	Chad Laing
	David Lapen

Powered by Editorial Manager® and ProduXion Manager® from Aries Systems Corporation

	Carson K. Leung
	Oliver Lung
**Order of Authors Secondary Information:**	
**Additional Information:**	
**Question**	**Response**
Are you submitting this manuscript to a special series or article collection?	No
**Experimental design and statistics**<br><br>Full details of the experimental design and statistical methods used should be given in the Methods section, as detailed in our Minimum Standards Reporting Checklist. Information essential to interpreting the data presented should be made available in the figure legends.<br><br>Have you included all the information requested in your manuscript?	Yes
**Resources**<br><br>A description of all resources used, including antibodies, cell lines, animals and software tools, with enough information to allow them to be uniquely identified, should be included in the Methods section. Authors are strongly encouraged to cite Research Resource Identifiers (RRIDs) for antibodies, model organisms and tools, where possible.<br><br>Have you included the information requested as detailed in our Minimum Standards Reporting Checklist?	Yes
**Availability of data and materials**<br><br>All datasets and code on which the conclusions of the paper rely must be either included in your submission or deposited in publicly available repositories (where available and ethically appropriate), referencing such data using	Yes

a unique identifier in the references and in the “Availability of Data and Materials” section of your manuscript.<br><br>Have you have met the above requirement as detailed in our Minimum Standards Reporting Checklist?	
GigaScience has policies and guidelines in place for the use of generative AI-writing tools such as ChatGPT. If you have used such writing tools to assist with writing the manuscript this must be declared and cited in the text. Authors should not list AI-writing tools and other AI-assisted technologies as an author or co-author and should acknowledge that they are fully responsible for text generated or refined by AI-writing tools.<p><br><br>A summary of use (particularly in the introduction or among methods) needs to be included at the end of the paper, and the outputs should also be included as a supplementary file hosted in GigaDB or other open repositories. Please read our guidelines for more information. <p><br><br>By submitting to GigaScience, you are aware of the journal's AI-writing tools policy, and if you have declared use of such tools below, you have acknowledged this where appropriate in your manuscript and have made a summary of use and outputs available. <p><br><b>AI-assisted writing tools have been used in the preparation of this manuscript?	No

Powered by Editorial Manager® and ProduXion Manager® from Aries Systems Corporation

WaveSeekerNet: Accurate Prediction of Influenza A Virus Subtypes and Host Source Using Attention-Based Deep Learning

Hoang-Hai Nguyen[1,2#], Josip Rudar[2,4#], Nathaniel Lesperance[4,6], Oksana Vernygora[2], Graham W. Taylor[5,6], Chad Laing[2], David Lapen[7], Carson K. Leung[1], Oliver Lung[2,3]

[1] Department of Computer Science, University of Manitoba, Winnipeg, Manitoba, Canada.

[2] National Centre for Foreign Animal Disease, Canadian Food Inspection Agency, Winnipeg, Manitoba, Canada.

[3] Department of Biological Sciences, University of Manitoba, Winnipeg, Manitoba, Canada.

[4] Department of Integrative Biology & Centre for Biodiversity Genomics, University of Guelph, Guelph, Ontario, Canada.

[5] School of Engineering, University of Guelph, Guelph, Ontario, Canada.

[6] Vector Institute, Toronto, Ontario, Canada.

[7] Agriculture and Agri-Food Canada, Ottawa, Canada.

#Address correspondence to Hoang-Hai Nguyen (nguyen92@myumanitoba.ca) and Josip Rudar (joe.rudar@inspection.gc.ca)

Abstract

Background

Influenza A virus (IAV) poses a significant threat to animal health globally, with its ability to overcome species barriers and cause pandemics. Rapid and accurate prediction of IAV subtypes and host source is crucial for effective surveillance and pandemic preparedness. Deep learning has emerged as a powerful tool for analyzing viral genomic sequences, offering new ways to uncover hidden patterns associated with viral characteristics and host adaptation.

Findings

We introduce WaveSeekerNet, a novel deep learning model for accurate and rapid prediction of IAV subtypes and host source. The model leverages attention-based mechanisms and efficient token mixing schemes, including the Fast Fourier Transform and the Wavelet Transform, to capture intricate patterns within viral RNA and protein sequences. Extensive experiments on diverse datasets demonstrate WaveSeekerNet's superior performance compared to existing Transformer-only models. Notably, WaveSeekerNet achieves scores of up to the maximum 1.0 across all evaluation metrics, including F1-score (Macro Average), Balanced Accuracy and Matthews Correlation Coefficient (MCC), in subtype prediction, even for rare subtypes. Furthermore, WaveSeekerNet exhibits remarkable accuracy in distinguishing between human, avian, and other mammalian hosts. The ability of WaveSeekerNet to flag potential cross-species transmission events underscores its significant value for real-time surveillance and proactive pandemic preparedness efforts.

Conclusions

WaveSeekerNet's superior performance, efficiency, and ability to flag potential cross-species transmission events highlight its potential for real-time surveillance and pandemic preparedness. This model represents a significant advancement in applying deep learning for IAV classification and holds promise for future epidemiological and veterinary studies, and public health interventions.

Keywords: deep learning, fast fourier transform, wavelet transform, chaos game representation, influenza A virus, antigenic types, viral host source.

1 Introduction

Influenza viruses are a constant threat to public and animal health and a leading cause of acute respiratory diseases worldwide. Avian influenza is a contagious disease caused by infection with Influenza A virus (IAV), which naturally resides and circulates in waterfowl [1]. Host barriers prevent IAVs from freely infecting new non-avian hosts; however, cross-species transmission can occur when viruses evolve to overcome these barriers [2]. One crucial determinant of IAV infection is hemagglutinin (HA) receptor binding specificity. Human influenza virus strains prefer to bind to α2,6-sialic acid linkages, whereas avian virus strains preferentially bind receptors of α2,3-sialic acid linkages [3]. IAV has a complex genome structure with eight negative-sense, single-stranded RNA gene segments: PB2, PB1, PA, HA, NP, NA, M, and NS [4]. The hemagglutinin (HA) and neuraminidase (NA) gene segments are two major determinants of IAV antigenicity and virulence. IAV is classified into subtypes based on HA and NA. There are 18 different HA subtypes (H1 through H18), 11 different NA subtypes (N1 through N11), and a possibility for new subtypes to be discovered, such as putative H19 [5], creating many possible

combinations, such as H5N1, H3N2, and H7N9. Since 1918, there have been four major influenza epidemics and pandemics caused by IAV: Spanish flu 1918 (H1N1), Asian flu 1957 (H2N2), Hong Kong flu 1968 (H3N2), and the 2009 swine flu pandemic (H1N1) [6,7]. Between 2003 and 2005, H5N1 IAV emerged in China and spread to other countries, causing widespread poultry outbreaks across Asia, Africa, the Middle East, and Europe [8,9]. In November 2021, a highly pathogenic avian influenza (HPAI) variant of H5N1 IAV emerged in domestic birds at a farm in Newfoundland, Canada, likely caused by transatlantic spread from Europe by wild birds [10]. Since its emergence, H5N1 IAV has circulated throughout North America. As of February 2025, this outbreak has affected nearly 14.5 million birds in Canada [11] and more than 162 million birds in the United States [12]. This has had a significant impact on poultry production and directly led to rising egg prices and food inflation in the United States [13]. The virus has also spread to mammals such as skunks, foxes, raccoons, dogs, and dairy cows, including humans [14,15]. Because of this widespread impact and potential for further spread, understanding and predicting the host source of IAV is crucial for active surveillance and our preparedness for future pandemics.

Although progress has been made in understanding IAV transmission, significant knowledge gaps remain [16,17]. Recently, machine learning (ML) and artificial intelligence (AI) are being used to analyze large-scale viral genomic sequences to facilitate a deeper understanding of the evolution and biology of pathogens. For example, machine learning algorithms like Random Forest, and Support Vector Machine (SVM) have been used to identify genomic patterns associated with expanded host range in severe acute respiratory syndrome coronavirus 2 (SARS-CoV-2) [18] and IAV [19–22]. However, increasing focus is being directed towards deep learning using artificial intelligence due to the flexibility in specifying models and ability to identify

complex relationships by weighing and transforming information contained within multiple genomic regions within a single model [23,24].

Ensemble learning has been successfully applied to supervised classification tasks in diverse fields [25,26]. Breiman (2001) demonstrated that this procedure is successful since decorrelation between members of the ensemble enables diverse learners to compensate for the errors of other members of the ensemble [27]. This observation was further developed in methods such as stacked generalization and voting classifiers, which exploit differences in the inductive biases of different machine learning algorithms to improve classification performance [28]. Deep learning has also leveraged the advantages of ensembles. For example, deep ensembles, which borrow from the work of Breiman (2001), have been used as an alternative to Bayesian Neural Networks to estimate prediction uncertainties [29,30]. Other innovations, such as dropout [31], act like ensembling by activating a different set of neurons with each training iteration. Finally, approaches such as multi-head attention [32] and Mixture-of-Experts layers [33] also contribute to achieving good generalization.

In this study, we present a new deep learning model, WaveSeekerNet, based on an ensemble of different efficient attention-like and feed-forward network mechanisms. WaveSeekerNet first splits image representations of RNA and protein-coding sequences into different ‘word’ patches known as tokens. Our ensemble attention-like mechanism, the WaveSeeker block, then aggregates information from each token, forming a kind of genomic signature allowing the modelling of underlying biological patterns and functional relationships tied to important outcomes such as the host source. WaveSeekerNet precisely classifies subtypes and predicts the host source of IAV by using the RNA sequences and protein-coding sequences of the HA and NA gene segments. Tests on held-out data reveal that WaveSeekerNet achieved state-

of-the-art performance. Furthermore, WaveSeekerNet successfully identified zoonosis, reverse zoonosis and enzootic strains.

2 Material and Methods

2.1 Influenza A Virus dataset and general workflow

IAV HA and NA RNA and protein-coding sequences were downloaded from EpiFlu GISAID in January and June 2024 [34] along with subtype, host and other available metadata. The EpiFlu GISAID database contains numerous identical sequences (Figure 1). For example, the HA segment of the avian H5N1 strain *A/goose/Zhejiang/727098/2014* (EPI681274) is identical to that of the swine H5N1 strain *A/swine/Zhejiang/SW57/2015* (EPI1600724). In this work, we chose to keep the earliest collected sequence and its associated metadata. Any identical sequences which were collected at a later time were removed from the analysis. After this step, an additional length and ambiguity filtering was performed. The length of the HA and NA segments from the *A/New York/392/2004* reference strain (H3N2 - HA segment: EPI79008, NA segment: EPI79013) was used as a baseline for identifying sequences that are either too short or too long. HA or NA sequences with a length at least 80% or at most 120% of the reference and which contained a maximum of 10 ambiguous sequence characters were included in the set of high-quality sequences. Any remaining sequence not meeting either of these criteria were put into a low-quality dataset if they had a minimum length of 1000nt or 350aa (for HA) and 850nt or 250aa (for NA), otherwise, they were excluded from analysis. We screened RNA sequences in the collected datasets using the VADR (Viral Annotation DefineR) tool [35] and reverse complemented any sequences which were not in the 5’ to 3’ direction.

To predict future viral characteristics, we split the high-quality and low-quality sequences temporally. High-quality sequences collected before January 1st 2020 were reserved for training, while those collected since 2020 were used for evaluating generalization performance. This strategy ensured that highly similar strains were not found in both training and testing sets. Due to overrepresentation of some HA and NA subtypes and underrepresentation of others, we down-sampled overrepresented subtypes (defined as having more than 6,000 sequences) to 6,000 sequences per subtype. Examples of overrepresented subtypes include H1 (56,593 RNA sequences), H3 (59,661 RNA sequences), N1 (47,592 RNA Sequences), and N2 (58,750 RNA Sequences). Rare subtypes, defined as having fewer than 600 sequences, were up-sampled to 600 sequences per subtype. Notably, subtypes H17 (2 sequences), H18 (1 sequence), N10 (2 sequences), and N11 (1 sequence), are extremely rare and were only included in the training data. Maintaining sequence diversity during training is crucial to prevent model bias, particularly towards rare subtypes. While the approximate 6000-sequence provides sufficient diversity for popular subtypes [36], the 600-sequence for rare subtypes requires up-sampling to mitigate this risk. We performed down- and up-sampling (copying the original multiple times) using the resample function from scikit-learn (v.1.5.1). This resulted in a final training set consisting of 18 hemagglutinin and 11 neuraminidase subtypes.

For the host source prediction dataset, sequences were also labelled according to the source from which they were isolated. In this work, we grouped sequences into three major host groups: Humans, Avian (e.g., falcon, turkey, goose), and Non-human Mammals (e.g., swine, horses, dogs). Since the human and avian hosts accounted for the majority of sequences, these groups were down-sampled to 16,000 sequences per group while all Non-human Mammals sequences were used. We used the high and low-quality sequences collected after December 31, 2019, to evaluate the

performance of models. We also identified the subset of strains where both an HA and NA sequence were present and used these sequences together for host source prediction (Figure 2a, Table S5). The data distribution used in this study is shown in Supplementary Tables S1-5 and Figure 1 illustrates the major steps in this study.

Figure 1: The general workflow consists of several steps. First, we retrieved sequences from EpiFlu GISAID along with subtype and host information. We removed duplicated sequences and kept one sequence with the earliest collected sequence and its associated metadata. Next, the sequences underwent quality control and distribution into training and test sets. Finally, the quality-controlled sequences were encoded into the form of images (2D matrix) using One-hot encoding and Frequency Chaos Game Representation (FCGR). The 2D image forms of sequences were then used to train the models and make the predictions.

2.2 Transforming sequences into feature vectors

To enable the neural network models to learn and recognize patterns of RNA and protein-coding sequences, we employed two methods to encode sequences into the form of images: Frequency Chaos Game Representation (FCGR) and One-hot encoding. The information content within nucleic acid and protein sequences can be viewed as a signal which itself can be transformed into an image and used as input for machine learning algorithms [37–39]. There have been numerous examples in the literature which have taken the Chaos Game Representation (CGR) approach. In the early 1990s, for example, the CGR of nucleic acids was used to visualize the structure of DNA sequences [40]. The CGR maps a DNA/RNA sequence S onto the unit square using Supplementary Algorithm S1.

An extension of CGR, the Frequency Chaos Game Representation [41,42], subdivides the CGR into $2^k \times 2^k$ sub-squares. The count of points within each sub-square represents the *k*-mer from the original sequence S. Due to variations in sequence length within the collected data, *k*-mer frequencies within the FCGRs require standardization for comparison. To address this, we applied the standardization method proposed by Wang et al. [43] (Equation 1) to create the standardized FCGR $\bar{A}$, which was used for our analysis of IAV RNA sequences. FCGRs were created using

the ‘complexcgr’ package version 0.8.0 (https://github.com/AlgoLab/complexCGR) in a Python 3.12 environment.

$$\bar{A} = \frac{4^k}{\sum_{i=1}^{k}\sum_{j=1}^{k} a_{i,j}} \quad (1)$$

where A is kth-order FCGR of sequence S, $a_{i,j}$ denotes the elements of A.

For protein sequences, we used the one-hot encoding to transform sequences into representations suitable for machine learning applications. This method has been used successfully to study influenza viruses, rotavirus A, rabies lyssavirus and predict, with some accuracy, host-range, antigenic types and pathogenicity [24,44]. Each character in the protein sequence is encoded as a 21-dimensional vector via one-hot encoding. Aggregation of these vectors forms a 2D matrix encoding an amino-acid sequence. For example, A (Alanine) can be encoded as (1, 0, 0, …, 0, 0, 0), C (Cysteine) as (0, 1, 0, …, 0, 0, 0), D (Aspartate) as (0, 0, 1, …, 0, 0, 0) and X as (0, 0, 0, …, 0, 0, 1). We zero-padded the C-terminal end of each HA and NA polypeptide sequence to ensure that the inputs to each deep learning model have the same length.

3 Model Architecture

We propose a deep learning network called WaveSeekerNet, as illustrated in Figure 2. The approach is based on attention-like architecture [32] and Vision Transformer [45]. Figure 2a presents the overall structure of WaveSeekerNet. The first step of our approach is to split the FCGRs and One-hot encodings of input sequences into ‘word’ patches (tokens). These ‘word’ patches are then flattened and linearly projected using noisy linear layers to create an embedding of each patch E_{patch} [46]. A learnable sinusoidal positional encoding based on positional encoding P in Transformer model [32] is then added to E_{patch} according to Equation 2 to create the final

patch embedding $E_{in} \in \mathbb{R}^{b \times n \times d}$ [47], where b, n, and d are the batch, token, and hidden (embedding) size, respectively. For multiple channels, WaveSeekerNet concatenates the embedding of the channels along the token dimension. The final embedding E_{in} is fed to the transformer-like WaveSeeker block (see section 3.1). After passing through the WaveSeeker block, the transformed embeddings are pooled using Global Expectation Pooling [48] and sent to the classification head. Here either the traditional feed-forward network or the Kolmogorov-Arnold Networks (KAN) [49] is used to perform classification.

$$E_{in} = E_{patch} + \sigma(PW_1 + b_1)W_2 + b_2 \quad (2)$$

Figure 2: (a) The overall structure of WaveSeekerNet. (b) The WaveSeeker block contains token mixing schemes: The Fourier Transform, The Wavelet Transform, gMLP. Other key components include a Sparsely Gated Multi-Head Mixture-of-Experts layer (MH-SMoE), StarNet. (c) The modified StarNet with an MLP-Mixer layer and Noisy Factorized Linear layers. (d) The token mixing schemes using the Fast Fourier Transform. (e) The token mixing schemes using the Wavelet Transform.

3.1 WaveSeeker block

The key component of WaveSeekerNet is the WaveSeeker block (Figure 2b). We incorporate significant changes in this block, which take advantage of lessons learned from ensemble approaches. In this study, the attention mechanism [32] is replaced by three token mixing approaches: the Fourier Transform, the Wavelet Transform, and the gating Multilayer Perceptron (gMLP) architecture [50]. The outputs of these token mixing blocks are concatenated along the hidden dimension and then merged using a modified version of the recently released StarNet [51]. In the StarNet block (Figure 2c), we replace depth-wise convolution with an MLP-Mixer layer [52] and apply noisy linear layers [46] before start operation (element-wise multiplication). To improve the capacity of the network, the traditional feed-forward layer of Vision Transformer [45] is replaced by a Sparsely Gated Multi-Head Mixture-of-Experts layer (MH-SMoE) [33,53,54].

3.1.1 Fast Fourier Transform block

A significant amount of effort has been directed towards developing alternatives to the self-attention mechanism due to its high computational and memory cost [52,55,56]. Research conducted thus far strongly suggests that algorithms which efficiently share information between tokens is required to develop an efficient alternative to the transformer; one of the first works to identify this approach is the FNET [55]. At the heart of this alternative to the transformer is the Fourier Transform. FNET applies a 2D discrete Fourier Transform (DFT) to the input embeddings. This transform block mixes information from each of the tokens and during training the model learns the weights associated with the best combination of tokens which minimizes the loss function. In image processing, the Fast Fourier Transform (FFT) is commonly used to compute the DFT, transforming an image into its frequency domain. Low frequencies represent global patterns while high frequencies correspond to abrupt changes, such as edges, which provide more details in the image. The 2D DFT and inverse DFT (iDFT) of an image array can be calculated using Equations 3 and 4:

$$F(x,y) = \sum_{m=0}^{M-1}\sum_{n=0}^{N-1} f(m,n) e^{-j2\pi(\frac{x}{M}m+\frac{y}{N}n)} \quad (3)$$

$$f(m,n) = \frac{1}{MN}\sum_{x=0}^{M-1}\sum_{y=0}^{N-1} F(x,y)\, e^{j2\pi(\frac{x}{M}m+\frac{y}{N}n)} \quad (4)$$

where $F(x,y)$ is the function to represent the image in the frequency domain, $f(m,n)$ is a pixel at position (m,n) in the spatial domain, $M \times N$ represents the dimension of the image.

In this study, we modified the original FNET architecture [55] to design an additional token mixing scheme as illustrated in Figure 2d. Tokens are first mixed by applying the 2D DFT to transform sequence embedding $E_{fft\_in} \in \mathbb{R}^{b \times n \times d}$ into frequency coefficients. Like FNET, we only keep the real part of the transform result. Since the FFT of a real signal is Hermitian-

symmetric, we omit the negative frequencies, producing $X_{fft} \in \mathbb{R}^{b \times n \times (d/2+1)}$. Subsequently, X_{fft} is projected back to the embedding dimension d and reshaped to create multi-head $X_{MHA} \in \mathbb{R}^{(b*h) \times n \times 32}$, where the number of heads $h = d/32$. We then employ an efficient attention with linear complexities [56] on X_{MHA} to capture intricate interactions within the frequency space. The output of this efficient attention mechanism is then enhanced with a dropout, RMS normalization [57], skip connections, yielding $X_{MHA}^{out} \in \mathbb{R}^{b \times n \times d}$. Next, we merge heads of X_{MHA}^{out} into $X_{merge} \in \mathbb{R}^{b \times n \times (d/2+1)}$. This prepares the representation for subsequent processing and transformation back into the spatial domain.

It is likely that the information needed to reconstruct each embedding from X_{merge} using the iDFT will be concentrated within the low-frequency components. This presents itself as an opportunity to promote sparsity and regularize this block of the network. This can be accomplished by applying a soft-thresholding operation on X_{merge} (Equations 4-6) [58]. Finally, tokens are de-mixed by inverse FFT (iFFT), which transforms the thresholded X_{thresh} (Equation 6) back to the spatial domain $X_{ifft} \in \mathbb{R}^{b \times n \times d}$. X_{ifft} is then scaled so that each element is between +5 and -5, which is then passed through the feed-forward network followed by dropout along the token dimension. Supplementary Algorithm S2 presents the Pseudo-code of the FFT block.

$$X_{shrunk} = X_{merge} - \arctan(X_{merge}) \quad (4)$$

$$X_{gate} = \{1 \; if \; |X_{shrunk}| - \lambda > 0 \; else \; 0\} \quad (5)$$

$$X_{thresh} = X_{shrunk} * X_{gate} \quad (6)$$

3.1.2 Wavelet Transform block

While the Fourier Transform captures global frequency information, it does not localize these frequencies within a sequence. The Wavelet Transform can be used to address this shortcoming since this transformation is capable of identifying where frequency components occur within a signal. This is particularly useful in sequence analysis since biological signals found in nucleic acid and protein sequences change over time [59]; thus, the Wavelet Transform can be applied to locate relevant frequency components in local regions of the sequence. In image processing, the discrete Wavelet Transform (DWT) is used to divide spatial information present in the image into low- and high-frequency components corresponding to approximation and detail coefficients, respectively. In the Wavelet Transform, a signal is convolved with bandpass filters or mother wavelets $\psi_{a,b}(t)$ (Equation 7) where a, b determine the scale and location of the wavelet, respectively [60,61]. The wavelet will be squeezed shorter in time (or space) when a is decreasing, capturing high-frequency components. In contrast, increasing the value of a will stretch the wavelet and capture low-frequency components. In terms of location, the wavelet shifts to the left with decreasing b and to the right with increasing b.

$$\psi_{a,b}(t) = \frac{1}{\sqrt{a}}\psi\left(\frac{t-b}{a}\right) \qquad a, b \in \mathbb{R} \qquad (7)$$

Formally, let sequence embedding $E_{dwt\_in} \in \mathbb{R}^{b \times n \times d}$, we perform initial projection on E_{dwt_in}, then split embedding dimension into h heads, where $h = d/32$, creating initial input $X_{init} \in \mathbb{R}^{b \times h \times n \times 32}$ for the Wavelet Transform. Next, we apply 2D DWT using the Pytorch Wavelets package version 1.3.0 [62] to transform the input space X_{init} to the Wavelet coefficients. Concretely, the Wavelet Transform applies low-pass and high-pass filters to transform X_{init} into Y_L and Y_H subbands. Y_L refers to the approximation coefficients that reflect the overall structure of

the input space at coarse-grained level. Y_H represents detail coefficients at fine-grained level, which is a single stacked tensor of Y_{LH} (horizontal detail), Y_{HL} (vertical detail), Y_{HH} (diagonal detail). Instead of using an efficient attention layer, as in the FFT block, we further processed Y_L and Y_H by the StartNet layer (Figure 2c). Like the FFT block, we apply the skip-connections, RMS normalization in the Wavelet coefficient space, and shrink high-frequency components in the Y_H subbands using Equations 4-6. The processed Y_L and Y_H are then used to transform feature maps back to the spatial domain using inverse DWT (iDWT), which is then passed through a feed-forward network followed by dropout along the token dimension. Supplementary Algorithm S3 presents the pseudo-code of the Wavelet Transform block.

4 Implementation and evaluation methods

4.1 Model training and testing

In this study, we compared the prediction performance of WaveSeekerNet with that of established Transformer-only models. Xu et al. [23,63] previously employed Transformer-only models to predict IAV host source and antigenic types. These models were among the most effective applications of machine learning algorithms for predicting IAV host source and subtypes. As part of our comparative analysis, we also explored the integration of the FNET architecture [55] into Transformer-only models, replacing the conventional multi-head attention mechanism. Table 1 presents the hyperparameter settings used to train each deep learning model. We also introduce a new activation function, ErMish (Equation 8), which allows for a greater range of negative activations. This activation function uses a learnable parameter, α, to adjust how positive and negative input values influence the magnitude and sign of the function's output:

$$ErMish(x) = \frac{3x}{2} erf\left(\alpha + \frac{\tanh(\ln(1 + e^{x}))}{\sqrt{2}}\right) \quad (8)$$

We used 10-fold stratified cross-validation to assess the generalization performance of models. We trained WaveSeekerNet using a composite loss function (Equation 9). The first part of the composite loss function is the cross-entropy loss function. This function is typically used to measure how much the prediction from the model, $\hat{y}$, deviates from the expected classification outcome, y. The second part of the loss function, the router z-loss, penalizes large logits during routing into the MoE network with the goal of forcing the model to balance the number of tokens routed to each expert. The last part of the loss function encourages the selection of specific activation functions within each of the KAN networks used in the classification head by reducing the impact of unnecessary activation functions on the final output of the KAN layer [49]. See Supplementary Algorithms S4 and S5 for details.

$$Loss(y, \hat{y}, x, w) = CE\ Loss(y, \hat{y}) + Z\ Loss(x) + KAN\ Loss(w) \quad (9)$$

We observed that Transformer-only models required more time to converge than our model; therefore, Transformer-only models were trained with 150 epochs, while WaveSeekerNet was trained with only 35 epochs. The weights of the last epoch were used for testing, and the reported scores represent the mean across the 10 folds. We conducted ablation studies to evaluate the impact of different hyperparameters (Table 1) on WaveSeekerNet's performance. We used the 'baycomp' package version 1.0.3 (https://github.com/janezd/baycomp) [64] to compare the generalization performance of WaveSeekerNet with various hyperparameters.

Table 1: The hyperparameter settings used by WaveSeekerNet and Transformer-only models during cross-validation.

Model	Hyperparameter Settings	Explanation of Parameters

WaveSeekerNet	Default Settings (Baseline Model):<br>• *use_fft* = True<br>• *use_wavelets* = True<br>• *used_gmlp* = True<br>• *wavelet_names* = “sym4”<br>• *emb_dim* = 64<br>• *final_hidden_size* = 24<br>• *use_kan* = True<br>• *use_smoe* = True<br>• *use_gc* = True<br>• *use_lookahead* = True<br>• *activation* = ErMish<br><br>Common Settings:<br>• *batch size* = 256<br>• *epochs* = 35<br><br>Settings for FCGR representation of RNA sequences (apply for all hyperparameters, models):<br>• *k*-mer = 6 (FCGR array size of 64 × 64)<br>• *patch size* = (4, 4)<br><br>Settings for One-hot encoding data of protein sequences (apply for all hyperparameters, models):<br>• *patch size* = (3, 21)	• *use_fft* – Specify whether the Fast Fourier Transform block is used in the WaveSeeker block.<br>• *use_wavelets* – Specify whether the Wavelet Transform block is used in the WaveSeeker block.<br>• *use_gmlp* – Specify whether the gMLP block is used in the WaveSeeker block.<br>• *wavelet_names* – Specify wavelet family for the Wavelet transform, it will be used when *use_wavelets* = True.<br>• *emb_dim* – The embedding dimension of the model.<br>• *final_hidden_size* – The size of the penultimate layer in the classification head.<br>• *use_kan* – Specify whether the KAN network is used as the classification head.<br>• *use_smoe* – Specify whether the model will use a MH-SMoE in the FFN layer of the WaveSeeker block.<br>• *use_gc* – Specify whether the Gradient Centralization is used in the optimizer [65].<br>• *use_lookahead* – Specify whether lookahead is used in the optimizer [66].<br>• *activation* – Specify activation for model (ErMish, Mish [67], GELU [68], ReLU [69]).<br>• *k*-mer – the *k*th-order of FCGR.<br>• *patch size* – size of ‘word’ patches.
Transformer-only using FNET	• *emb_dim* = 64, 128<br>• *activation* = ReLU<br>• *epochs* = 150<br>• *batch size* = 256	• *emb_dim* – The embedding dimension of the model.
Transformer-only using conventional multi-head attention (MHA)	• *emb_dim* = 64, 128<br>• *nhead* = 4<br>• *activation* = ReLU<br>• *epochs* = 150<br>• *batch size* = 256	• *emb_dim* – The embedding dimension of the model.<br>• *nhead* – The number of heads in the multi-head attention

4.2 Sequence similarity search methods

We evaluated the performance of deep learning models against BLAST, a widely used sequence similarity search method in computational biology and bioinformatics. Comparisons to BLAST search served as a sanity check to ensure that our model, and other deep learning methods, did not suffer from systemic failures and worked as expected. For protein sequence-based subtype prediction, BLASTp (v2.13.0) [70] with options '-num_alignments 100 -evalue 1e-5'. The BLASTp database was built using the same training splits of the high-quality pre-2020 dataset used for model training. This was done to ensure that results using sequence similarity search methods would be comparable. The query then consisted of every strain found in the post-2020 high- and low-quality testing data. The top hit was determined to be the hit with the highest bit score after filtering for hits with at least 85% identity and an alignment length of at least 50. For query sequences that did not have any hits, we set an arbitrary subtype (1-18 for H subtype, 1-11 for N subtype) as the prediction.

For subtype prediction using RNA sequences, we used VADR [35], a tool for viral sequence classification and annotation. VADR uses a model library from a subset of references to classify and annotate virus sequences. We used the *v-annotate.pl* VADR script (v1.6.4) with influenza models (v1.6.3-2) [71] for subtype classification.

4.3 Analysis of the ongoing H5Nx outbreak in North America

To investigate WaveSeekerNet's ability to identify the most probable source of a transmission event, we used data from the ongoing H5Nx outbreak in North America as a test case. We identified a subset of our data consisting of 1,659 unique HA RNA sequences from various H5Nx strains circulating in North America in 2023 and 2024. These strains represent a recent snapshot of the ongoing H5Nx Avian Influenza outbreak. To support the predictions made by

WaveSeekerNet, we performed a phylogenetic analysis of the H5Nx virus dataset. Sequences were aligned with MAFFT version v7.520 [72,73]. Time-calibrated phylogenetic analysis was performed in BEAST v1.10.4 [74] using a coalescent Bayesian skyline model [75,76] and a general time-reversible substitution model with the across-site rate heterogeneity sampled from a gamma distribution with four discrete categories and uncorrelated relaxed log-normal molecular clock [77]. Two independent runs were performed with a chain length of 50 million generations each and parameter values sampled every 1,000 generations. Stationarity and convergence of independent runs were assessed in Tracer v1.7 [78]. The LogCombiner module in the BEAST software was used to remove burn-in fraction and combine log and tree files. The maximum clade credibility tree with median node heights was produced using TreeAnnotator [74]. Phylogenetic trees were visualized using FigTree v.1.4.3 (https://github.com/rambaut/figtree) and Inkscape (https://inkscape.org/).

4.4 Evaluation Metrics

Evaluation metrics used in this study include F1-score (Macro Average), Balanced Accuracy, and Matthews Correlation Coefficient (MCC). Since the dataset is unbalanced and we treat all classes equally regardless of their support values, thus we used F1-score (Macro Average) to compare the performance between methods. This metric is computed by taking the arithmetic mean of F1-score (Equation 10) of all classes. This measurement approach is appropriate since we are interested in assessing the performance across the diverse range of subtypes and host categories in our data.

The equations of F1-score for each class, Balanced Accuracy and MCC are defined as follows:

$$F1 - score = \frac{2*TP}{2*TP+FP+FN} \quad (10)$$

$$Balanced\ Accuracy = \frac{1}{2}\left(\frac{TP}{TP+FN} + \frac{TN}{TN+FP}\right) \quad (11)$$

$$MCC = \frac{TP*TN-FP*FN}{\sqrt{(TP+FP)(TP+FN)(TN+FP)(TN+FN)}} \quad (12)$$

where TP, FP, TN, and FN stand for True Positive, False Positive, True Negative and False Negative, respectively.

5 Results

5.1 WaveSeekerNet is robust to changes in hyperparameter settings

We conducted initial ablation studies to evaluate the impact of various hyperparameters, as illustrated in Table 1, on WaveSeekerNet's performance. For example, we disabled one or two token mixing schemes and used various activation functions. The experimental results of host source prediction, presented in Supplementary Tables S6, S7 and Supplementary Figures S1, S2, S3, demonstrate WaveSeekerNet's robustness to changes in hyperparameter settings under this testing regime. While some hyperparameter choices did influence performance, the baseline WaveSeekerNet model consistently demonstrated strong performance. This model of choice, using all 3 token mixing schemes (FFT, Wavelet Transform and gMLP blocks), ranked at or near the top across various data representations and datasets. When the baseline model did rank lower, the generalization performance of the alternative models was often similar to that of the baseline. Our results also show that disabling any of the three token mixing schemes resulted in a decrease in generalization performance in most RNA sequence tests, while yielding mixed results in protein sequence tests. Generally, empirical evidence from these tests indicated that the probability of

modifications to the baseline settings are unlikely to result in improvements in classification performance (Supplementary Tables S6, S7).

WaveSeekerNet's performance with the Wavelet Transform block was particularly impactful when trained on FCGR representation of RNA sequences (Supplementary Table S6 and Supplementary Figures S1a, S1b, S2a, S2b, S3a, S3b). The Wavelet Transform block improved the WaveSeekerNet's performance significantly. For example, when tested with HA RNA sequences, using gMLP-only, FFT and gMLP, and FFT-only token mixing schemes, WaveSeekerNet achieved F1-score (Macro Average) of 0.778, 0.749 and 0.811, respectively (Supplementary Table S6). While using all 3 token mixing schemes (the baseline model), Wavelet Transform-only, Wavelet Transform and gMLP, FFT and Wavelet Transform achieved F1-score (Macro Average) of 0.958, 0.96, 0.962 and 0.971, respectively.

Lookahead optimizer was another hyperparameter which influenced generalization performance. Disabling this optimizer consistently degraded WaveSeekerNet's performance across various data representations and datasets (Supplementary Tables S6, S7 and Supplementary Figures S1, S2, S3). For instance, when assessing the host source prediction using the FCGRs of HA segments (Supplementary Table S6), the F1-score (Macro Average) were 0.748±0.305 and 0.53±0.231 for high- and low-quality datasets, respectively, when Lookahead optimizer was disabled. In the baseline model, where Lookahead optimizer is enabled, the F1-score (Macro Average) in these tests were 0.958±0.023 and 0.707±0.068, respectively. Gradient centralization was also important, but primarily impacted results in tests using FCGRs. Disabling gradient centralization resulted in up to 4% decrease in F1-score (Macro Average) (Supplementary Table S6 and Supplementary Figure S1a). Our use of the ErMish activation positively impacted WaveSeekerNet's performance, particularly in the protein sequence datasets (Supplementary

Table S7). Alternative activations, such as Mish, GELU, and ReLU, often had a probability of less than 0.5 of outperforming the baseline model. Finally, we observed that alternative configurations, such as disabling KAN layers and MH-SMoE, negatively impacted generalization performance. This was evident in both RNA and protein sequence datasets, where the baseline model without KAN or MH-SMoE generally underperformed the baseline model (Supplementary Tables S6, S7).

5.2 WaveSeekerNet can predict Influenza HA and NA subtypes with high accuracy

We compared WaveSeekerNet's performance in predicting HA subtypes with Transformer-only models, VADR and BLASTp (Supplementary Figure S4 and Figure 3). When tested on high-quality datasets of RNA and protein sequences, WaveSeekerNet achieved a minimum score of 0.97 across all evaluation metrics and hyperparameters (Supplementary Figures S4a and S4c). Notably, when trained WaveSeekerNet using FCGR representation of RNA sequences (Figures 3a, 3b and Supplementary Table S8), WaveSeekerNet obtained an F1-score (Macro Average) of 1.0 and 0.977 for high-quality and low-quality datasets, respectively. In contrast, Transformer-only models achieved maximum F1-score (Macro Average) of 0.804 and 0.425 for high-quality and low-quality datasets of RNA sequences, respectively. Compared with sequence similarity search methods, WaveSeekerNet's performance was comparable to VADR (Figures 3a, 3b) and BLASTp (Figures 3c, 3d). Notably, when tested on both the high-quality and low-quality datasets of RNA sequences, VADR produced an F1-score (Macro Average) of 1.0.

Figure 3: Results of HA subtype prediction when tested the best-performing WaveSeekerNet models, Transformer-only models. The performance of baseline WaveSeekerNet is also shown as a point of reference. The boxen plots of Balanced Accuracy, F1-score (Macro Average), and Matthews Correlation Coefficient (MCC) are reported for the high (a) and low (b) quality FCGR representation of RNA sequences. The boxen plots of scores for tests on the dataset constructed from high-quality and low-quality one-hot encoded protein sequences are reported in panels (c) and (d), respectively. Horizontal dash-lines present the F1-score (Macro Average) for VADR and BLASTp. The performance of the WaveSeekerNet models shows little variance and overlaps with that of VADR.

When classifying NA subtypes (Supplementary Figures S5, S6), the results mirrored the strong performance observed in HA subtype prediction. WaveSeekerNet achieved a minimum score of 0.991 across all evaluation metrics and hyperparameters on the high-quality datasets of RNA and protein sequences (Supplementary Figures S5a, S5c), with an exception of No Lookahead hyperparameter. Disabling Lookahead optimizer, the WaveSeekerNet only achieved an F1-score (Macro Average) of 0.912 on high-quality dataset of RNA sequences (Supplementary Figure S5a). WaveSeekerNet also obtained an F1-score (Macro Average) of 1.0 on high-quality dataset of RNA sequences (Supplementary Figures S5a). When tested on low-quality datasets of RNA and protein sequences (Supplementary Figures S6b, S6d and Supplementary Table S9), Transformer-only models still performed the worst, with a maximum F1-score (Macro Average) of 0.679. In contrast, WaveSeekerNet outperformed BLASTp and slightly underperformed VADR, maintaining an F1-score (Macro Average) of up to 0.951 and 1.0 when tested with low-quality datasets of RNA and protein sequences, respectively.

5.3 WaveSeekerNet accurately identifies the host source of using Influenza A virus consensus sequences

Figures 4, 5 compare the best-performing WaveSeekerNet models with that of Transformer-only models when tested on the HA segment and the combined of HA and NA segments. When tested on the high-quality datasets of the HA segment, the best-performing WaveSeekerNet model achieved a minimum score of 0.95 across all evaluation metrics (Figures 4a, 4c). In contrast, the Transformer-only models achieved maximum F1-score (Macro Average) of 0.92 and 0.91 for RNA and protein sequences, respectively. However, when evaluated on high-quality datasets of the combined HA and NA segments (Figures 5a, 5c) and the NA segment

(Supplementary Figures S7a, S7c), the best-performing Transformer-only models were comparable to WaveSeekerNet.

A substantial difference in performance was observed when evaluating low-quality datasets derived from protein sequences. WaveSeekerNet significantly surpassed the performance of Transformer-only models by up to 43% in terms of F1-score (Macro Average) and by up to 65% in terms of MCC on the combined HA and NA segments (Figure 5d) and the HA segment (Figure 4d).

Figure 4: Results of host source prediction using the HA segment when tested the best-performing WaveSeekerNet models, Transformer-only models. The performance of baseline WaveSeekerNet is also shown as a point of reference. The boxen plots of Balanced Accuracy, F1-score (Macro Average), and Matthews Correlation Coefficient (MCC) are reported for the high (a) and low (b) quality FCGR representation of RNA sequences. The boxen plots of scores for tests on the dataset constructed from high-quality and low-quality one-hot encoded protein sequences are reported in panels (c) and (d), respectively.

Figure 5: Results of host source prediction using the combined HA and NA segments (2 channels) when tested the best-performing WaveSeekerNet models, Transformer-only models. The performance of baseline WaveSeekerNet is also shown as a point of reference. The boxen plots of Balanced Accuracy, F1-score (Macro Average), and Matthews Correlation Coefficient (MCC) are reported for the high (a) and low (b) quality FCGR representation of RNA sequences. The boxen plots of scores for tests on the dataset constructed from high-quality and low-quality one-hot encoded protein sequences are reported in panels (c) and (d), respectively.

5.4 Host prediction discrepancies can carry important information about recent transmission events.

In the previous sections, we demonstrated that WaveSeekerNet can predict subtypes and host sources with high accuracy. Prediction discrepancies were noted and likely carry important information concerning recent transmission events since it implies that the extracted consensus sequence signature more closely resembles the signature from a different host. WaveSeekerNet produced multiple lines of evidence to support this hypothesis. For example, two H5N1 IAVs, *A/CastillaLaMancha/3739/2022* (EPI_ISL_15542438) and *A/CastillaLaMancha/3869/2022*

(EPI_ISL_16813290), were isolated in humans but were classified as having an avian origin by WaveSeekerNet. Upon further investigation, we found that the Spanish Influenza National Reference Laboratory (NRL) linked these samples to an outbreak from a poultry farm where workers were likely infected by hens [79]. In another case, our approach identified the HA and NA genes from a sample *A/China/ZMD-22-2/2022* (EPI_ISL_15613648) as having an avian origin. This sample was isolated from a patient carrying an avian H3N8 virus [80]. In another instance, a sample isolated in March 2024, *A/Vietnam/KhanhhoaRV1-005/2024* (EPI_ISL_19031556), was correctly identified by WaveSeekerNet as having an avian origin. Contact tracing and viral characterization results revealed that the 21-year-old man from Khanh Hoa Province, Vietnam, was exposed to wild birds and subsequently infected with an avian influenza H5N1 virus [81,82].

In addition, our results suggest that discrepancies could also identify reverse zoonotic transmission events. For example, our model classified *A/swine/North Carolina/A02751330/2022* (EPI_ISL_16891306), *A/swine/Ohio/A02751292/2022* (EPI_ISL_16891307), *A/swine/Cambodia/PFC37/2021* (EPI_ISL_17885993), and *A/swine/Cambodia/PFC33/2020* (EPI_ISL_17886005) as human sequences. This could be interpreted as these strains containing a genomic signature resembling other human sequences. Subsequent phylogenetic analyses and molecular characterization demonstrated that these sequences are closely related to those circulating in nearby human populations, suggesting a human-to-swine transmission event [83,84]. Supplementary Tables S10, S11, S12 provide additional details of discrepancies identified by our model.

5.5 Spillover events from the ongoing H5Nx Avian Influenza outbreak are flagged by WaveSeekerNet

WaveSeekerNet successfully identified the most probable animal source of transmission in 1,659 sequences collected from the ongoing H5Nx outbreaks in North America. The model predicted 99.9% of the sequences were of avian origin. For example, infections of mammals with the HPAI clade 2.3.4.4b H5N1, H5N5 viruses were recently detected [14,15,85]. These include H5N5 cases collected in Canada (e.g., *A/Raccoon/PEI/FAV-0199-1/2023* and *A/Striped_Skunk/PEI/FAV-0210-1/2023)*, H5N1 cases in dairy cows in the United State (e.g., *A/dairy_cow/Colorado/24_018028-008/2024*, and *A/dairy_cow/Colorado/24_018028-013/2024*). Furthermore, a phylogenetic analysis supported the predictions for other H5N1 cases (e.g., humans, cat, dairy cow, goat, swine). This analysis revealed that these samples are nested within avian sub-clades (Figure 6). Additionally, the collection sites of these samples are within the same geographical areas, providing additional evidence for a relationship between the avian strains and closely related strains isolated from non-avian hosts. Details of the sequences, predictions made by WaveSeekerNet are available in Supplementary Table S13.

Figure 6: Pruned time-calibrated phylogeny of 1,659 HA RNA sequences collected from the recent H5N1 IAV outbreaks in North America. Different spillover events from the ongoing outbreak of HPAI H5N1 into mammalian hosts are highlighted in blue, orange, and green. WaveSeekerNet identified these strains as having an avian origin, a discrepancy which is supported by the phylogenetic analysis. Bars at the nodes indicate 95% highest posterior density of the estimated node dates.

6 Discussion

This work presents a transformer-like architecture, WaveSeekerNet, inspired by ensemble learning, which can sufficiently capture and generalize information found, in relatively simple sequence transformations and encodings, into a coherent internal representation of human, avian or non-human mammal-adapted IAV irrespective of subtype. The exceptional accuracy of

WaveSeekerNet in predicting IAV subtypes and host source in both high- and low-quality test data underscores the role modern deep learning can play in rapid and effective influenza surveillance and characterization: especially with human activities such as hunting, agriculture, movement, and urbanization dramatically increasing interactions between humans and animal populations, subsequently increasing the risk of zoonotic transmission [86,87]. This increase in transmission risk is best exemplified by the emergence of the 2009 H1N1 IAV pandemic, which was a triple reassortant virus between human, avian, and swine strains [88], and the recent emergence of SARS-CoV-2 virus, which was associated with wildlife sold at the Huanan seafood market in Wuhan, China [89].

The approach presented here is advantageous since WaveSeekerNet was trained using simple sequence transformations and encodings. Simple transformations are effective since they are well studied and their applicability to problems such as these is known [24,37,38,44]. By utilizing these transformation methods, we can have greater confidence that any observed improvements can be attributed to our architectural choices rather than a lightly investigated and poorly understood choice of transformation. For example, the frequency of specific *k*-mers and codon pairs was found to be important for classifying potential hosts of H3Nx viruses [22]. Depending on the depth of the FCGR, these frequencies and features are a natural feature of the representation. Furthermore, by using the FCGR, we are also able to capture the unique fractal properties within the genome [42,90,91]. Together, this allows the model to learn from an information-dense set of features, which we demonstrate with our exceptional performance in subtype and host source prediction. Finally, since these transformations can be created by applying existing tools and do not require any specialized knowledge to use, the workflow needed to run an analysis is greatly simplified. This is a critical requirement in diagnostic settings where the

generation of accurate and reproducible reports for a timely and effective response to a disease outbreak is needed.

Time-based data partitioning as a departure from the more traditional approach of random data partitioning allows us to better estimate how well WaveSeekerNet will perform since new viral strains are constantly emerging, and there may not be points of comparison available in datasets at the time of sequencing. Generalizing when trained on imbalanced data is also important because recognizing rarer, emerging variants is essential for successful surveillance and characterization, which in turn informs public health responses. Often, this information is discarded, or analyses do not fully consider information from rarer variants and noisy data. This can potentially result in an overestimation of generalization performance. For example, Xu et al. considered rare subtypes (e.g., H15, H17, H18, N10 and N11) together as a single group when testing their Transformer-only models and used a weighted one-vs-all strategy to produce F1-score [23,63]. However, this strategy is biased towards good performance in majority classes and, as we show, can result in overly optimistic performance scores (Supplementary Tables S8, S9). The ability to handle imbalanced data and noise and generalize from a small number of samples is vital in real-world scenarios where information is often missing. Approaches, such as a WaveSeekerNet, which are better able to handle this data can enhance the ability of public health authorities to detect and properly respond to outbreaks caused by emerging or rare strains.

The WaveSeekerNet's ability to accurately classify host source, even when trained on diverse subtypes, suggests that the model is learning underlying genomic signatures linked to host adaptation. This is best exemplified with our model's ability to accurately identify the correct subtype and host source in temporally separated data. Furthermore, we provide evidence that shows that discrepancies between predictions and known host source likely indicate higher

similarity with genome signatures from another host. This could mean that viruses have had limited time to evolve within and adapt to a new host. For example, our phylogenetic analyses of H5Nx viruses associated with the ongoing HPAI outbreak in North America reveals that the genomic signature found in sequences in non-human mammals is very similar to that of circulating avian strains, suggesting a recent introduction into these non-human mammal group. This observation is likely influenced by the fact that influenza viruses tend to accumulate mutations over time that optimize their fitness within a particular host species, leading to distinct genomic signatures associated with different hosts (e.g., avian, swine, humans) [92–94]. Therefore, if a virus jumps to a new host and retains a signature similar to its previous host, it suggests the jump was recent and there has not been sufficient time for the virus to undergo extensive mutations needed to adapt fully to the new host environment.

WaveSeekerNet's design is based on an ensemble attention-like mechanism which involves splitting viral sequences into ‘word’ patches and mixing them. This process allows the model to develop an internal representation of each of the classes and leverages this representation to make predictions. Further work is needed to identify ‘word’ patches strongly impacting predictions, which can reveal important sequence properties underpinning viral adaptation and transmission in new hosts. This could be achieved by using Explainable AI (XAI) techniques, like SHAP (SHapley Additive exPlanations) [95], that measure how impactful a feature is on the output of a trained model. In this work, ‘word’ patches with high SHAP values can be interpreted as specific genetic determinants of host adaptation. Previously, SHAP values were used to find specific mutations in the genome of SARS-CoV-2 potentially associated with adaptation in deer and mink hosts [18]. While a similar approach can be taken here, additional care must be taken to understand how much each feature’s SHAP value changes with different initial parameterizations

of WaveSeekerNet. Therefore, an optimal strategy which identifies maximally relevant features inextricably involves minimizing the impact of noise arising from differences in the initial state of WaveSeekerNet. Once found, the insights revealed using these tools could deepen our understanding of viral evolution and biology in novel hosts and provide a secondary, independent source of evidence to support and complement traditional phylogenetic analyses and experimentation using animal models.

To enhance the robustness and capacity of our model, we incorporated some new ideas that yielded promising results. Additionally, replacing the traditional feed-forward network within the WaveSeeker block with the Sparse Mixture-of-Experts network, also led to a demonstrable improvement in the performance of WaveSeekerNet. This work also demonstrated the potential of the KAN, a recently developed alternative to traditional feed-forward networks, in improving the identification of biological sequences in the majority of tests. The applicability of KAN and Sparse Mixture-of-Experts network extends beyond the identification and characterization of IAV, and we believe that these approaches can be effectively applied to broader taxonomic identification problems such as assigning taxonomic labels (e.g., species, genus, family) to DNA and RNA sequences generated by high-throughput sequencing technologies. While the baseline WaveSeekerNet model has exhibited strong performance, the impact of specific hyperparameters, such as the Wavelet Transform block, KAN, Sparse Mixture-of-Experts, optimizer, and the choice of activation functions, highlights the importance of careful model design and hyperparameter tuning. Future work should focus on refining these hyperparameters and further exploring additional architectural enhancements to improve WaveSeekerNet's accuracy and generalizability. Moreover, incorporating XAI techniques is essential to better understand the model's decision-making processes, ultimately facilitating more effective model refinement and optimization.

WaveSeekerNet represents an advancement in applying deep learning for influenza virus classification and host source prediction. Its accuracy, efficiency, and potential for revealing novel biological insights position it as a valuable tool for future influenza surveillance and pandemic preparedness. We will integrate WaveSeekerNet into CFIA-NCFAD/nf-flu (https://github.com/CFIA-NCFAD/nf-flu) [96], an existing IAV analysis workflow at the Canadian Food Inspection Agency (CFIA) - National Centre For Foreign Animal Disease (NCFAD). This integration will enable a more comprehensive analysis of IAV, ultimately contributing to enhanced surveillance and pandemic preparedness in Canada. Furthermore, our work highlights the crucial role of deep learning in analyzing medically important data, as deep learning excels at discovering and utilizing long-range patterns in high-quality data for improved prognostic accuracy [97,98].

7 Limitations and Future Work

While this study presents a significant advancement in IAV prediction, there are limitations to address in future research:

- *Explainable AI*: Using explainable AI approaches would enhance the interpretability of WaveSeekerNet's predictions, providing insights into the specific sequence features driving its classifications. This would also provide mutations associated with host-prediction which could be verified experimentally.
- *Complex Transformations*: Exploring more complex input transformations that incorporate amino acid properties could further improve the model's ability to capture subtle differences between strains and hosts.

- *Species-Level Breakdown*: Expanding host source prediction to a species-level breakdown within avian and non-human mammals would offer more granular insights for targeted surveillance and control measures.

By addressing these limitations, future iterations of WaveSeekerNet can provide even more powerful tools for understanding and combating IAV.

Figure Legends

Figure 1: The general workflow consists of several steps. First, we retrieved sequences from EpiFlu GISAID along with subtype and host information. We removed duplicated sequences and kept one sequence with the earliest collected sequence and its associated metadata. Next, the sequences underwent quality control and distribution into training and test sets. Finally, the quality-controlled sequences were encoded into the form of images (2D matrix) using One-hot encoding and Frequency Chaos Game Representation (FCGR). The 2D image forms of sequences were then used to train the models and make the predictions.

Figure 2: (a) The overall structure of WaveSeekerNet. (b) The WaveSeeker block contains token mixing schemes: The Fourier Transform, The Wavelet Transform, gMLP. Other key components include a Sparsely Gated Multi-Head Mixture-of-Experts layer (MH-SMoE), StarNet. (c) The modified StarNet with an MLP-Mixer layer and Noisy Factorized Linear layers. (d) The token mixing schemes using the Fast Fourier Transform. (e) The token mixing schemes using the Wavelet Transform.

Figure 3: Results of HA subtype prediction when tested the best-performing WaveSeekerNet models, Transformer-only models. The performance of baseline WaveSeekerNet is also shown as a point of reference. The boxen plots of Balanced Accuracy, F1-score (Macro Average), and Matthews Correlation Coefficient (MCC) are reported for the high (a) and low (b) quality FCGR representation of RNA sequences. The boxen plots of scores for tests on the dataset constructed from high-quality and low-quality one-hot encoded protein sequences are reported in panels (c) and (d), respectively. Horizontal dash-lines present the F1-score (Macro Average) for VADR and BLASTp. The performance of the WaveSeekerNet models shows little variance and overlaps with that of VADR.

Figure 4: Results of host source prediction using the HA segment when tested the best-performing WaveSeekerNet models, Transformer-only models. The performance of baseline WaveSeekerNet is also shown as a point of reference. The boxen plots of Balanced Accuracy, F1-score (Macro Average), and Matthews Correlation Coefficient (MCC) are reported for the high (a) and low (b) quality FCGR representation of RNA sequences. The boxen plots of scores for tests on the dataset constructed from high-quality and low-quality one-hot encoded protein sequences are reported in panels (c) and (d), respectively.

Figure 5: Results of host source prediction using the combined HA and NA segments (2 channels) when tested the best-performing WaveSeekerNet models, Transformer-only models. The performance of baseline WaveSeekerNet is also shown as a point of reference. The boxen plots of Balanced Accuracy, F1-score (Macro Average), and Matthews Correlation Coefficient (MCC) are reported for the high (a) and low (b) quality FCGR representation of RNA sequences. The boxen plots of scores for tests on the dataset constructed from high-quality and low-quality one-hot encoded protein sequences are reported in panels (c) and (d), respectively.

Figure 6: Pruned time-calibrated phylogeny of 1,659 HA RNA sequences collected from the recent H5N1 IAV outbreaks in North America. Different spillover events from the ongoing outbreak of HPAI H5N1 into mammalian hosts are highlighted in blue, orange, and green. WaveSeekerNet identified these strains as having an avian origin, a discrepancy which is supported by the phylogenetic analysis. Bars at the nodes indicate 95% highest posterior density of the estimated node dates.

Additional Files

Figure S1: The generalization performance of WaveSeekerNet for host source prediction was evaluated on the HA segment using various hyperparameter settings. The Balanced Accuracy, F1-score (Macro Average), and Matthews Correlation Coefficient (MCC) are reported for high- (a) and low- (b) quality FCGR representation of RNA sequences. Scores for tests on the dataset constructed from high-quality and low-quality one-hot encoded protein sequences are reported in panels (c) and (d), respectively.

Figure S2: The generalization performance of WaveSeekerNet for host source prediction was evaluated on the NA segment using various hyperparameter settings. The Balanced Accuracy, F1-score (Macro Average), and Matthews Correlation Coefficient (MCC) are reported for high- (a) and low- (b) quality FCGR representation of RNA sequences. Scores for tests on the dataset constructed from high-quality and low-quality one-hot encoded protein sequences are reported in panels (c) and (d), respectively.

Figure S3: The generalization performance of WaveSeekerNet for host source prediction was evaluated on the combined HA and NA segments (2 channels) using various hyperparameter settings. The Balanced Accuracy, F1-score (Macro Average), and Matthews Correlation Coefficient (MCC) are reported for high- (a) and low- (b) quality FCGR representation of RNA sequences. Scores for tests on the dataset constructed from high-quality and low-quality one-hot encoded protein sequences are reported in panels (c) and (d), respectively.

Figure S4: The generalization performance of WaveSeekerNet and Transformer-only for HA subtype prediction using various hyperparameter settings. The Balanced Accuracy, F1-score (Macro Average), and Matthews Correlation Coefficient (MCC) are reported for high- (a) and low- (b) quality FCGR representation of RNA sequences. Panels (c) and (d) show scores for high- and low-quality one-hot encoded protein sequences, respectively. WaveSeekerNet and Transformer-only using various hyperparameter settings are labeled in Black and Blue, respectively. The F1-score (Macro Average) for VADR and BLASTp are shown as red horizontal lines.

Figure S5: The generalization performance of WaveSeekerNet and Transformer-only for NA subtype prediction using various hyperparameter settings. The Balanced Accuracy, F1-score (Macro Average), and Matthews Correlation Coefficient (MCC) are reported for high- (a) and low- (b) quality FCGR representation of RNA sequences. Panels (c) and (d) show scores for high- and low-quality one-hot encoded protein sequences, respectively. WaveSeekerNet and Transformer-only using various hyperparameter settings are labeled in Black and Blue, respectively. The F1-score (Macro Average) for VADR and BLASTp are shown as red horizontal lines.

Figure S6: Results of NA subtype prediction when tested the best-performing WaveSeekerNet models, Transformer-only models. The performance of baseline WaveSeekerNet is also shown as a point of reference. The boxen plots of Balanced Accuracy, F1-score (Macro Average), and Matthews Correlation Coefficient (MCC) are reported for the high (a) and low (b) quality FCGR representation of RNA sequences. The boxen plots of scores for tests on the dataset constructed from high-quality and low-quality one-hot encoded protein sequences are reported in panels (c) and (d), respectively. Horizontal dash-lines present the F1-score (Macro Average) for VADR and BLASTp. The performance of the WaveSeekerNet models shows little variance and overlaps with that of VADR.

Figure S7: Results of host source prediction using the NA segment when tested the best-performing WaveSeekerNet models, Transformer-only models. The performance of baseline WaveSeekerNet is also shown as a point of reference. The boxen plots of Balanced Accuracy, F1-score (Macro Average), and Matthews Correlation Coefficient (MCC) are reported for the high (a) and low (b) quality FCGR representation of RNA sequences. The boxen plots of scores for tests on the dataset constructed from high-quality and low-quality one-hot encoded protein sequences are reported in panels (c) and (d), respectively.

Table S1: The data distribution of HA Subtypes. The numbers in the parentheses are the number of sequences before up-sampling or down-sampling.

Table S2: The data distribution of HA sequences used for host source prediction. The numbers in the parentheses are the number of sequences before up-sampling or down-sampling.

Table S3: The data distribution of NA Subtypes. The numbers in the parentheses are the number of sequences before up-sampling or down-sampling.

Table S4: The data distribution of NA sequences used for host source prediction. The numbers in the parentheses are the number of sequences before up-sampling or down-sampling.

Table S5: The data distribution of the combined HA and NA sequences used for host source prediction. The numbers in the parentheses are the number of sequences before up-sampling or down-sampling.

Table S6: The experimental results of host source prediction when testing the trained WaveSeekerNet using FCGR representation of RNA sequences.

Table S7: The experimental results of host source prediction when testing the trained WaveSeekerNet using One-hot encoding representation of protein sequences.

Table S8: The report of HA subtype-specific performance when tested models using FCGR representation of RNA sequences. The F1-scores were obtained using the best-performing WaveSeekerNet models and Transformer-only models. Models were trained to recognize the 18 HA subtypes (18 classes in the training data). Asterisks (*) indicate the absence of data for specific subtypes in the testing datasets. For these subtypes, we report an F1-score of N/A. F1-scores were calculated after 10 cross-validation folds and are presented for both the high-quality and low-quality datasets.

Table S9: The report of NA subtype-specific performance when tested models using FCGR representation of RNA sequences. The F1-scores were obtained using the best-performing WaveSeekerNet models and Transformer-only models. Models were trained to recognize the 11 NA subtypes (11 classes in the training data). Asterisks (*) indicate the absence of data for specific subtypes in the testing datasets. For these subtypes, we report an F1-score of N/A. F1-scores were calculated after 10 cross-validation folds and are presented for both the high-quality and low-quality datasets.

Table S10: The report of host discrepancies identified by WaveSeekerNet when tested on the HA segment.

Table S11: The report of host discrepancies identified by WaveSeekerNet when tested on the NA segment.

Table S12: The report of host discrepancies identified by WaveSeekerNet when tested on the combined HA and NA segments.

Table S13: The report of host source identified by WaveSeekerNet when tested on 1,659 HA RNA sequences from the ongoing H5Nx outbreaks in North America.

Algorithm S1: Transform each sequence into a chaos game representation.

Algorithm S2: Pseudo-code for the FFT block

Algorithm S3: Pseudo-code for the Wavelet Transform block

Algorithm S4: Calculation of the Router Z-loss

Algorithm S5: Calculation of the KAN Regularization Loss

Funding

H-H.N., J.R., C.L., O.V., and O.L. are supported by the Canadian Safety and Security Program (CSSP), grant CSSP-2022-CP-2538 (CSSP-2023-CP-2620) and Artificial Intelligence for front-line laboratories: preparing for high-consequence pathogens to reduce their risk). J.R., D.L., C.L., and O.L. are supported by the CSSP grant CSSP-2023-CP-2620 (Pilot Pan-Canadian Surveillance

for Mammalian Viral Pathogens Using Hematophagic Organisms and Environmental Samples). H-H.N. and C.K.L. are partially supported by Natural Sciences and Engineering Research Council of Canada (NSERC) and University of Manitoba. G.W.T. and N.L. are supported by the INSPIRE (Integrated Network for the Surveillance of Pathogens: Increasing REsilience and capacity in Canada's pandemic response) project funded through the Canada Biomedical Research Fund (grant no. CBRF2-2023-00008), the Biomedical Research Infrastructure Fund, and the Ontario Research Fund. This research was supported, in part, by the Province of Ontario and the Government of Canada through the Canadian Institute for Advanced Research (CIFAR), and companies sponsoring the Vector Institute (https://vectorinstitute.ai/partnerships/current-partners/).

Author's Contributions

H-H.N. designed the models, wrote the code/manuscript, prepared data, trained models, performed experiments and completed the data analysis. J.R. designed models, wrote the code, reviewed/edited the manuscript, mentored and provided critical feedback. O.V. performed phylogenetic analysis and reviewed/edited the manuscript. N.L. demonstrated the viability of the FCGR. O.L, C.K.L, and G.W.T supervised the study, reviewed/edited the manuscript. D.L., C.L. reviewed/edited the manuscript. All authors contributed to finalizing the manuscript.

Acknowledgements

We thank Peter Kruczkiewicz, Cass Erdelyan, Dr. Anthony Signore and Dr. Yohannes Berhane for valuable comments and feedback.

Competing Interests

The authors have declared that no competing interests exist.

Data Availability

The metadata, experimental results, training logs are available at project home page (https://github.com/nhhaidee/WaveSeekerNet), the RNA and protein sequences of HA and NA segments of IAV can be downloaded from EpiFlu GISAID (https://gisaid.org/) after creating account and accepting the terms of use.

Availability of Source Code and Requirements

- Project Name: WaveSeekerNet: Accurate Prediction of Influenza A Virus Subtypes and Host Source Using Attention-Based Deep Learnings
- Project home page: https://github.com/nhhaidee/WaveSeekerNet
- Operating System(s): e.g, Platform independent
- Programming Language: Python 3.12.5
- Other requirements: Python 3.12+, pytorch 2.4.1, pytorch-optimizer 3.1.1, pytorch-wavelets 1.3.0, scikit-learn 1.5.1, complexcgr 0.8.0, seaborn 0.13.2, matplotlib 3.9.1, pyfastx 2.1.0, pandas 2.2.2, numpy 1.26.4, biopython 1.84, baycomp 1.0.3.
- License: MIT

References

[1] Webster RG, Bean WJ, Gorman OT, Chambers TM, Kawaoka Y. Evolution and ecology of influenza A viruses. Microbiol Rev 1992;56:152–79. https://doi.org/10.1128/mr.56.1.152-179.1992.

[2] Kuiken T, Holmes EC, McCauley J, Rimmelzwaan GF, Williams CS, Grenfell BT. Host Species Barriers to Influenza Virus Infections. Science 2006;312:394–7. https://doi.org/10.1126/science.1122818.

[3] Imai M, Kawaoka Y. The role of receptor binding specificity in interspecies transmission of influenza viruses. Curr Opin Virol 2012;2:160–7. https://doi.org/10.1016/j.coviro.2012.03.003.

[4] Bouvier NM, Palese P. The biology of influenza viruses. Vaccine 2008;26:D49–53. https://doi.org/10.1016/j.vaccine.2008.07.039.

[5] Fereidouni S, Starick E, Karamendin K, Di Genova C, Scott SD, Khan Y, et al. Genetic characterization of a new candidate hemagglutinin subtype of influenza A viruses. Emerg Microbes Infect 2023;12:2225645. https://doi.org/10.1080/22221751.2023.2225645.

[6] Kilbourne ED. Influenza Pandemics of the 20th Century. Emerg Infect Dis 2006;12:9–14. https://doi.org/10.3201/eid1201.051254.

[7] Al Hajjar S, McIntosh K. The first influenza pandemic of the 21st century. Ann Saudi Med 2010;30:1–10. https://doi.org/10.4103/0256-4947.59365.

[8] Smith GJD, Fan XH, Wang J, Li KS, Qin K, Zhang JX, et al. Emergence and predominance of an H5N1 influenza variant in China. Proc Natl Acad Sci 2006;103:16936–41. https://doi.org/10.1073/pnas.0608157103.

[9] Emergence and Evolution of H5N1 Bird Flu | Avian Influenza (Flu) 2024. https://archive.cdc.gov/www_cdc_gov/flu/avianflu/communication-resources/bird-flu-origin-infographic.html (accessed November 4, 2024).

[10] Caliendo V, Lewis NS, Pohlmann A, Baillie SR, Banyard AC, Beer M, et al. Transatlantic spread of highly pathogenic avian influenza H5N1 by wild birds from Europe to North America in 2021. Sci Rep 2022;12:11729. https://doi.org/10.1038/s41598-022-13447-z.

[11] Canadian Food Inspection Agency. Status of ongoing avian influenza response by province 2023. http://inspection.canada.ca/en/animal-health/terrestrial-animals/diseases/reportable/avian-influenza/latest-bird-flu-situation/status-ongoing-response (accessed February 6, 2025).

[12] CDC. USDA Reported H5N1 Bird Flu Detections in Poultry. Avian Influenza Bird Flu 2025. https://www.cdc.gov/bird-flu/situation-summary/data-map-commercial.html (accessed February 19, 2025).

[13] Bohannon M. Egg Shortage: As Prices Climb 15%, Stores Nationwide Begin To Ration Egg Purchases. Forbes n.d. https://www.forbes.com/sites/mollybohannon/2025/02/14/egg-shortage-as-prices-climb-15-stores-nationwide-begin-to-ration-egg-purchases/ (accessed February 19, 2025).

[14] Erdelyan CNG, Kandeil A, Signore AV, Jones MEB, Vogel P, Andreev K, et al. Multiple transatlantic incursions of highly pathogenic avian influenza clade 2.3.4.4b A(H5N5) virus

into North America and spillover to mammals. Cell Rep 2024;43:114479. https://doi.org/10.1016/j.celrep.2024.114479.

[15] Caserta LC, Frye EA, Butt SL, Laverack M, Nooruzzaman M, Covaleda LM, et al. Spillover of highly pathogenic avian influenza H5N1 virus to dairy cattle. Nature 2024;634:669–76. https://doi.org/10.1038/s41586-024-07849-4.

[16] Sempere Borau M, Stertz S. Entry of influenza A virus into host cells — recent progress and remaining challenges. Curr Opin Virol 2021;48:23–9. https://doi.org/10.1016/j.coviro.2021.03.001.

[17] Aiello AE, Coulborn RM, Aragon TJ, Baker MG, Burrus BB, Cowling BJ, et al. Research findings from nonpharmaceutical intervention studies for pandemic influenza and current gaps in the research. Am J Infect Control 2010;38:251–8. https://doi.org/10.1016/j.ajic.2009.12.007.

[18] Rudar J, Kruczkiewicz P, Vernygora O, Golding GB, Hajibabaei M, Lung O. Sequence signatures within the genome of SARS-CoV-2 can be used to predict host source. Microbiol Spectr 2024;12:e03584-23. https://doi.org/10.1128/spectrum.03584-23.

[19] Eng CL, Tong JC, Tan TW. Predicting host tropism of influenza A virus proteins using random forest. BMC Med Genomics 2014;7:S1. https://doi.org/10.1186/1755-8794-7-S3-S1.

[20] Eng CLP, Tong JC, Tan TW. Predicting Zoonotic Risk of Influenza A Viruses from Host Tropism Protein Signature Using Random Forest. Int J Mol Sci 2017;18:1135. https://doi.org/10.3390/ijms18061135.

[21] Yin R, Zhou X, Rashid S, Kwoh CK. HopPER: an adaptive model for probability estimation of influenza reassortment through host prediction. BMC Med Genomics 2020;13:9. https://doi.org/10.1186/s12920-019-0656-7.

[22] Alberts F, Berke O, Maboni G, Petukhova T, Poljak Z. Utilizing machine learning and hemagglutinin sequences to identify likely hosts of influenza H3Nx viruses. Prev Vet Med 2024;233:106351. https://doi.org/10.1016/j.prevetmed.2024.106351.

[23] Xu Y, Wojtczak D. MC-NN: An End-to-End Multi-Channel Neural Network Approach for Predicting Influenza A Virus Hosts and Antigenic Types. SN COMPUT SCI 2023;4. https://doi.org/doi.org/10.1007/s42979-023-01839-5.

[24] Mock F, Viehweger A, Barth E, Marz M. VIDHOP, viral host prediction with deep learning. Bioinformatics 2021;37:318–25. https://doi.org/10.1093/bioinformatics/btaa705.

[25] Sarmah U, Borah P, Bhattacharyya DK. Ensemble Learning Methods: An Empirical Study. SN Comput Sci 2024;5:924. https://doi.org/10.1007/s42979-024-03252-y.

[26] Rudar J, Porter TM, Wright M, Golding GB, Hajibabaei M. LANDMark: an ensemble approach to the supervised selection of biomarkers in high-throughput sequencing data. BMC Bioinformatics 2022;23:110. https://doi.org/10.1186/s12859-022-04631-z.

[27] Breiman L. Random Forests. Mach Learn 2001;45:5–32. https://doi.org/10.1023/A:1010933404324.

[28] Wolpert DH. Stacked generalization. Neural Netw 1992;5:241–59. https://doi.org/10.1016/S0893-6080(05)80023-1.

[29] Lakshminarayanan B, Pritzel A, Blundell C. Simple and scalable predictive uncertainty estimation using deep ensembles. Proc. 31st Int. Conf. Neural Inf. Process. Syst., Red Hook, NY, USA: Curran Associates Inc.; 2017, p. 6405–16.

[30] Abe T, Buchanan EK, Pleiss G, Zemel R, Cunningham JP. Deep ensembles work, but are they necessary? Proc. 36th Int. Conf. Neural Inf. Process. Syst., Red Hook, NY, USA:

Curran Associates Inc.; 2022, p. 33646–60.

[31] Srivastava N, Hinton G, Krizhevsky A, Sutskever I, Salakhutdinov R. Dropout: A Simple Way to Prevent Neural Networks from Overfitting. J Mach Learn Res 2014;15:1929–58.

[32] Vaswani A, Shazeer N, Parmar N, Uszkoreit J, Jones L, Gomez AN, et al. Attention is all you need. Proc. 31st Int. Conf. Neural Inf. Process. Syst., Red Hook, NY, USA: Curran Associates Inc.; 2017, p. 6000–10.

[33] Shazeer N, Mirhoseini A, Maziarz K, Davis A, Le QV, Hinton GE, et al. Outrageously Large Neural Networks: The Sparsely-Gated Mixture-of-Experts Layer. CoRR 2017;abs/1701.06538.

[34] Shu Y, McCauley J. GISAID: Global initiative on sharing all influenza data – from vision to reality. Eurosurveillance 2017;22. https://doi.org/10.2807/1560-7917.ES.2017.22.13.30494.

[35] Schäffer AA, Hatcher EL, Yankie L, Shonkwiler L, Brister JR, Karsch-Mizrachi I, et al. VADR: validation and annotation of virus sequence submissions to GenBank. BMC Bioinformatics 2020;21:211. https://doi.org/10.1186/s12859-020-3537-3.

[36] Wang Y, Bao J, Du J, Li Y. Rapid Detection and Prediction of Influenza A Subtype using Deep Convolutional Neural Network based Ensemble Learning. Proc. 2020 10th Int. Conf. Biosci. Biochem. Bioinforma., New York, NY, USA: Association for Computing Machinery; 2020, p. 47–51. https://doi.org/10.1145/3386052.3386053.

[37] Avila Cartes J, Anand S, Ciccolella S, Bonizzoni P, Della Vedova G. Accurate and fast clade assignment via deep learning and frequency chaos game representation. GigaScience 2023;12:giac119. https://doi.org/10.1093/gigascience/giac119.

[38] Löchel HF, Eger D, Sperlea T, Heider D. Deep learning on chaos game representation for proteins. Bioinformatics 2020;36:272–9. https://doi.org/10.1093/bioinformatics/btz493.

[39] Chen D, Jacob L, Mairal J. Biological sequence modeling with convolutional kernel networks. Bioinformatics 2019;35:3294–302. https://doi.org/10.1093/bioinformatics/btz094.

[40] Jeffrey HJ. Chaos game representation of gene structure. Nucleic Acids Res 1990;18:2163. https://doi.org/10.1093/nar/18.8.2163.

[41] Almeida JS, Carriço JA, Maretzek A, Noble PA, Fletcher M. Analysis of genomic sequences by Chaos Game Representation. Bioinformatics 2001;17:429–37. https://doi.org/10.1093/bioinformatics/17.5.429.

[42] Deschavanne PJ, Giron A, Vilain J, Fagot G, Fertil B. Genomic signature: characterization and classification of species assessed by chaos game representation of sequences. Mol Biol Evol 1999;16:1391–9. https://doi.org/10.1093/oxfordjournals.molbev.a026048.

[43] Wang Y, Hill K, Singh S, Kari L. The spectrum of genomic signatures: from dinucleotides to chaos game representation. Gene 2005;346:173–85. https://doi.org/10.1016/j.gene.2004.10.021.

[44] Luo N, Wang X, Wang B, Meng R, Zhao Y, Chai Z, et al. Flu-CNN: predicting host tropism of influenza A viruses via character-level convolutional networks 2023:2023.08.28.23294703. https://doi.org/10.1101/2023.08.28.23294703.

[45] Dosovitskiy A, Beyer L, Kolesnikov A, Weissenborn D, Zhai X, Unterthiner T, et al. An Image is Worth 16x16 Words: Transformers for Image Recognition at Scale 2021. https://doi.org/10.48550/arXiv.2010.11929.

[46] Fortunato M, Azar MG, Piot B, Menick J, Osband I, Graves A, et al. Noisy Networks for Exploration 2019. https://doi.org/10.48550/arXiv.1706.10295.

[47] Wang G, Lu Y, Cui L, Lv T, Florencio D, Zhang C. A Simple yet Effective Learnable Positional Encoding Method for Improving Document Transformer Model. In: He Y, Ji H, Li S, Liu Y, Chang C-H, editors. Find. Assoc. Comput. Linguist. AACL-IJCNLP 2022, Online Only: Association for Computational Linguistics; 2022, p. 453–63. https://doi.org/10.18653/v1/2022.findings-aacl.42.

[48] Luo X, Tu X, Ding Y, Gao G, Deng M. Expectation pooling: an effective and interpretable pooling method for predicting DNA–protein binding. Bioinformatics 2020;36:1405–12. https://doi.org/10.1093/bioinformatics/btz768.

[49] Liu Z, Wang Y, Vaidya S, Ruehle F, Halverson J, Soljačić M, et al. KAN: Kolmogorov-Arnold Networks 2024. https://doi.org/10.48550/arXiv.2404.19756.

[50] Liu H, Dai Z, So D, Le QV. Pay Attention to MLPs. Adv. Neural Inf. Process. Syst., vol. 34, Curran Associates, Inc.; 2021, p. 9204–15.

[51] Ma X, Dai X, Bai Y, Wang Y, Fu Y. Rewrite the Stars 2024. https://doi.org/10.48550/arXiv.2403.19967.

[52] Tolstikhin I, Houlsby N, Kolesnikov A, Beyer L, Zhai X, Unterthiner T, et al. MLP-mixer: an all-MLP architecture for vision. Proc. 35th Int. Conf. Neural Inf. Process. Syst., Red Hook, NY, USA: Curran Associates Inc.; 2024, p. 24261–72.

[53] Wu X, Huang S, Wang W, Wei F. Multi-Head Mixture-of-Experts 2024. https://doi.org/10.48550/arXiv.2404.15045.

[54] Zoph B, Bello I, Kumar S, Du N, Huang Y, Dean J, et al. ST-MoE: Designing Stable and Transferable Sparse Expert Models 2022. https://doi.org/10.48550/arXiv.2202.08906.

[55] Lee-Thorp J, Ainslie J, Eckstein I, Ontañón S. FNet: Mixing Tokens with Fourier Transforms. CoRR 2021;abs/2105.03824.

[56] Shen Z, Zhang M, Zhao H, Yi S, Li H. Efficient Attention: Attention with Linear Complexities 2024. https://doi.org/10.48550/arXiv.1812.01243.

[57] Zhang B, Sennrich R. Root mean square layer normalization. Proc. 33rd Int. Conf. Neural Inf. Process. Syst., Red Hook, NY, USA: Curran Associates Inc.; 2019, p. 12381–92.

[58] Guibas J, Mardani M, Li Z, Tao A, Anandkumar A, Catanzaro B. Adaptive Fourier Neural Operators: Efficient Token Mixers for Transformers 2022. https://doi.org/10.48550/arXiv.2111.13587.

[59] Graur D, Li W-HL. Fundamentals of Molecular Evolution. Second Edition. Oxford, New York: Oxford University Press; 2000.

[60] Chen Y. An Introduction to Wavelet Analysis with Applications to Image and JPEG 2000. 2022 4th Int. Conf. Intell. Med. Image Process., New York, NY, USA: Association for Computing Machinery; 2022, p. 49–57. https://doi.org/10.1145/3524086.3524094.

[61] Soman KP, Ramachandran KI. Insight Into Wavelets From Theory To Practice 2Nd Ed. Prentice-Hall Of India Pvt. Limited; 2005.

[62] Cotter F. Uses of Complex Wavelets in Deep Convolutional Neural Networks 2020. https://doi.org/10.17863/CAM.53748.

[63] Xu Y, Wojtczak D. Dive into machine learning algorithms for influenza virus host prediction with hemagglutinin sequences. Biosystems 2022;220. https://doi.org/10.1016/j.biosystems.2022.104740.

[64] Benavoli A, Corani G, Demšar J, Zaffalon M. Time for a Change: a Tutorial for Comparing Multiple Classifiers Through Bayesian Analysis. J Mach Learn Res 2017;18:1–36.

[65] Yong H, Huang J, Hua X, Zhang L. Gradient Centralization: A New Optimization Technique for Deep Neural Networks. Comput. Vis. – ECCV 2020 16th Eur. Conf. Glasg.

UK August 23–28 2020 Proc. Part I, Berlin, Heidelberg: Springer-Verlag; 2020, p. 635–52. https://doi.org/10.1007/978-3-030-58452-8_37.

[66] Zhang MR, Lucas J, Hinton G, Ba J. Lookahead optimizer: k steps forward, 1 step back. Proc. 33rd Int. Conf. Neural Inf. Process. Syst., Red Hook, NY, USA: Curran Associates Inc.; 2019, p. 9597–608.

[67] Misra D. Mish: A Self Regularized Non-Monotonic Activation Function 2020. https://doi.org/10.48550/arXiv.1908.08681.

[68] Hendrycks D, Gimpel K. Gaussian Error Linear Units (GELUs) 2023. https://doi.org/10.48550/arXiv.1606.08415.

[69] Agarap AF. Deep Learning using Rectified Linear Units (ReLU) 2019. https://doi.org/10.48550/arXiv.1803.08375.

[70] Camacho C, Coulouris G, Avagyan V, Ma N, Papadopoulos J, Bealer K, et al. BLAST+: architecture and applications. BMC Bioinformatics 2009;10:421. https://doi.org/10.1186/1471-2105-10-421.

[71] Calhoun VC, Hatcher EL, Yankie L, Nawrocki EP. Influenza sequence validation and annotation using VADR. Database 2024;2024:baae091. https://doi.org/10.1093/database/baae091.

[72] Katoh K, Misawa K, Kuma K, Miyata T. MAFFT: a novel method for rapid multiple sequence alignment based on fast Fourier transform. Nucleic Acids Res 2002;30:3059–66. https://doi.org/10.1093/nar/gkf436.

[73] Katoh K, Standley DM. MAFFT Multiple Sequence Alignment Software Version 7: Improvements in Performance and Usability. Mol Biol Evol 2013;30:772–80. https://doi.org/10.1093/molbev/mst010.

[74] Suchard MA, Lemey P, Baele G, Ayres DL, Drummond AJ, Rambaut A. Bayesian phylogenetic and phylodynamic data integration using BEAST 1.10. Virus Evol 2018;4:vey016. https://doi.org/10.1093/ve/vey016.

[75] Drummond AJ, Rambaut A, Shapiro B, Pybus OG. Bayesian Coalescent Inference of Past Population Dynamics from Molecular Sequences. Mol Biol Evol 2005;22:1185–92. https://doi.org/10.1093/molbev/msi103.

[76] Drummond AJ, Nicholls GK, Rodrigo AG, Solomon W. Estimating Mutation Parameters, Population History and Genealogy Simultaneously From Temporally Spaced Sequence Data. Genetics 2002;161:1307–20. https://doi.org/10.1093/genetics/161.3.1307.

[77] Drummond AJ, Ho SYW, Phillips MJ, Rambaut A. Relaxed Phylogenetics and Dating with Confidence. PLOS Biol 2006;4:e88. https://doi.org/10.1371/journal.pbio.0040088.

[78] Rambaut A, Drummond AJ, Xie D, Baele G, Suchard MA. Posterior Summarization in Bayesian Phylogenetics Using Tracer 1.7. Syst Biol 2018;67:901–4. https://doi.org/10.1093/sysbio/syy032.

[79] Aznar E, Casas I, Praetorius AG, Ramos MJR, Pozo F, Moros MJS, et al. Influenza A(H5N1) detection in two asymptomatic poultry farm workers in Spain, September to October 2022: suspected environmental contamination. Eurosurveillance 2023;28:2300107. https://doi.org/10.2807/1560-7917.ES.2023.28.8.2300107.

[80] Bao P, Liu Y, Zhang X, Fan H, Zhao J, Mu M, et al. Human infection with a reassortment avian influenza A H3N8 virus: an epidemiological investigation study. Nat Commun 2022;13:6817. https://doi.org/10.1038/s41467-022-34601-1.

[81] Avian Influenza A(H5N1) – Viet Nam n.d. https://www.who.int/emergencies/disease-outbreak-news/item/2024-DON511 (accessed December 3, 2024).

[82] Authority EFS, European Centre for Disease Prevention and Control, Influenza EURL for A, Alexakis L, Fusaro A, Kuiken T, et al. Avian influenza overview March–June 2024. EFSA J 2024;22:e8930. https://doi.org/10.2903/j.efsa.2024.8930.
[83] Zeller MA, Carnevale de Almeida Moraes D, Ciacci Zanella G, Souza CK, Anderson TK, Baker AL, et al. Reverse zoonosis of the 2022–2023 human seasonal H3N2 detected in swine. Npj Viruses 2024;2:1–12. https://doi.org/10.1038/s44298-024-00042-4.
[84] Zeller MA, Ma J, Wong FY, Tum S, Hidano A, Holt H, et al. The genomic landscape of swine influenza A viruses in Southeast Asia. Proc Natl Acad Sci U S A 2023;120:e2301926120. https://doi.org/10.1073/pnas.2301926120.
[85] Baker AL, Arruda B, Palmer MV, Boggiatto P, Sarlo Davila K, Buckley A, et al. Dairy cows inoculated with highly pathogenic avian influenza virus H5N1. Nature 2024:1–8. https://doi.org/10.1038/s41586-024-08166-6.
[86] Soulsbury CD, White PCL. Human–wildlife interactions in urban areas: a review of conflicts, benefits and opportunities. Wildl Res 2015;42:541–53. https://doi.org/10.1071/WR14229.
[87] Richardson S, Mill AC, Davis D, Jam D, Ward AI. A systematic review of adaptive wildlife management for the control of invasive, non-native mammals, and other human–wildlife conflicts. Mammal Rev 2020;50:147–56. https://doi.org/10.1111/mam.12182.
[88] Garten RJ, Davis CT, Russell CA, Shu B, Lindstrom S, Balish A, et al. Antigenic and Genetic Characteristics of Swine-Origin 2009 A(H1N1) Influenza Viruses Circulating in Humans. Science 2009;325:197–201. https://doi.org/10.1126/science.1176225.
[89] Lu R, Zhao X, Li J, Niu P, Yang B, Wu H, et al. Genomic characterisation and epidemiology of 2019 novel coronavirus: implications for virus origins and receptor binding. The Lancet 2020;395:565–74. https://doi.org/10.1016/S0140-6736(20)30251-8.
[90] Anitas EM. Fractal Analysis of DNA Sequences Using Frequency Chaos Game Representation and Small-Angle Scattering. Int J Mol Sci 2022;23:1847. https://doi.org/10.3390/ijms23031847.
[91] Correia JP, Silva R, Anselmo DH a. L, Vasconcelos MS, da Silva LR. Multifractal Properties of Human Chromosome Sequences. Fractal Fract 2024;8:312. https://doi.org/10.3390/fractalfract8060312.
[92] Sanjuán R, Nebot MR, Chirico N, Mansky LM, Belshaw R. Viral Mutation Rates. J Virol 2010;84:9733–48. https://doi.org/10.1128/jvi.00694-10.
[93] Elderfield RA, Watson SJ, Godlee A, Adamson WE, Thompson CI, Dunning J, et al. Accumulation of Human-Adapting Mutations during Circulation of A(H1N1)pdm09 Influenza Virus in Humans in the United Kingdom. J Virol 2014;88:13269–83. https://doi.org/10.1128/jvi.01636-14.
[94] Taubenberger JK, Kash JC. Influenza Virus Evolution, Host Adaptation, and Pandemic Formation. Cell Host Microbe 2010;7:440–51. https://doi.org/10.1016/j.chom.2010.05.009.
[95] Lundberg SM, Lee S-I. A unified approach to interpreting model predictions. Proc. 31st Int. Conf. Neural Inf. Process. Syst., Red Hook, NY, USA: Curran Associates Inc.; 2017, p. 4768–77.
[96] Kruczkiewicz P, Nguyen HH, Meknas A, Erdelyan C, Lung O. CFIA-NCFAD/nf-flu: 3.7.0 2025. https://doi.org/10.5281/zenodo.14728750.
[97] Zhu W, Xie L, Han J, Guo X. The Application of Deep Learning in Cancer Prognosis Prediction. Cancers 2020;12:603. https://doi.org/10.3390/cancers12030603.
[98] Wekesa JS, Kimwele M. A review of multi-omics data integration through deep learning

approaches for disease diagnosis, prognosis, and treatment. Front Genet 2023;14. https://doi.org/10.3389/fgene.2023.1199087.

Figure 1

Click here to access/download;Figure;Figure 1.pdf

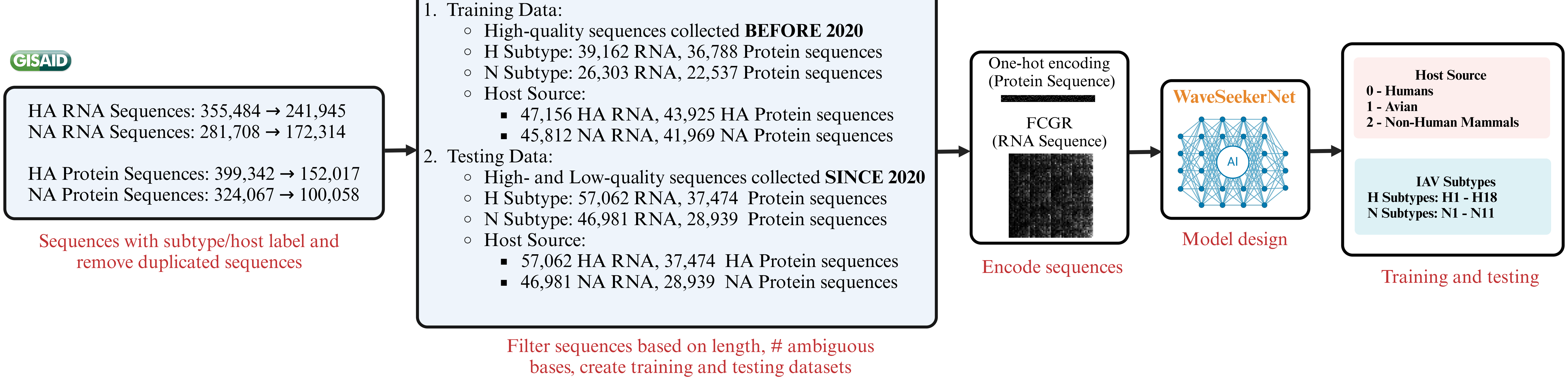

Figure 2

Click here to access/download;Figure;Figure 2.pdf

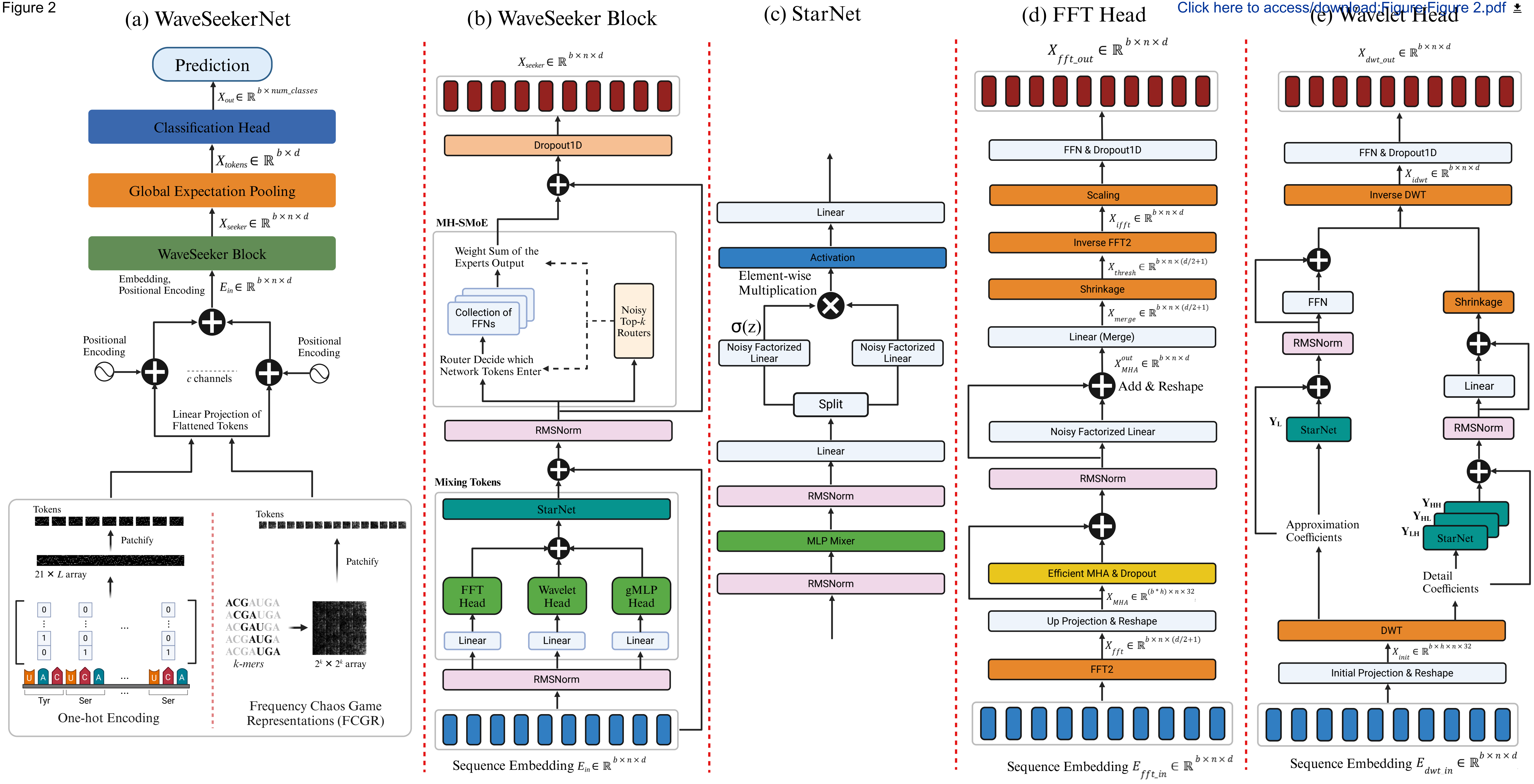

Figure 3

Click here to access/download;Figure;Figure 3.pdf

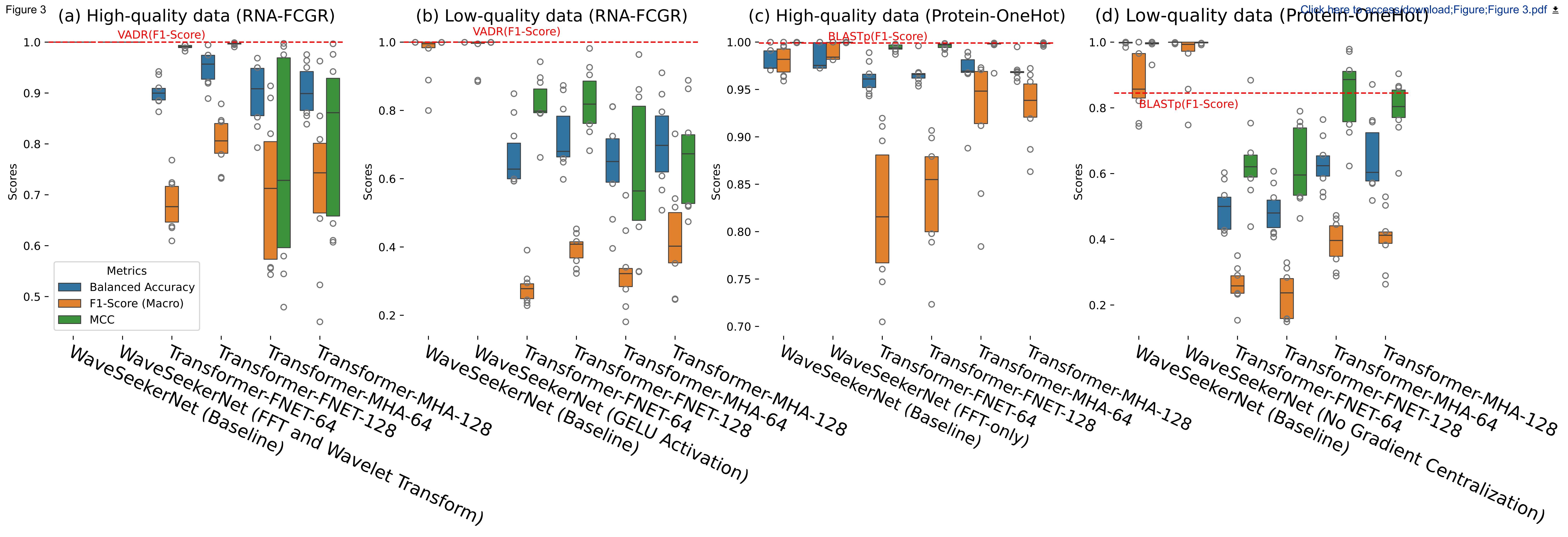

Figure 4

Click here to access/download;Figure;Figure 4.pdf

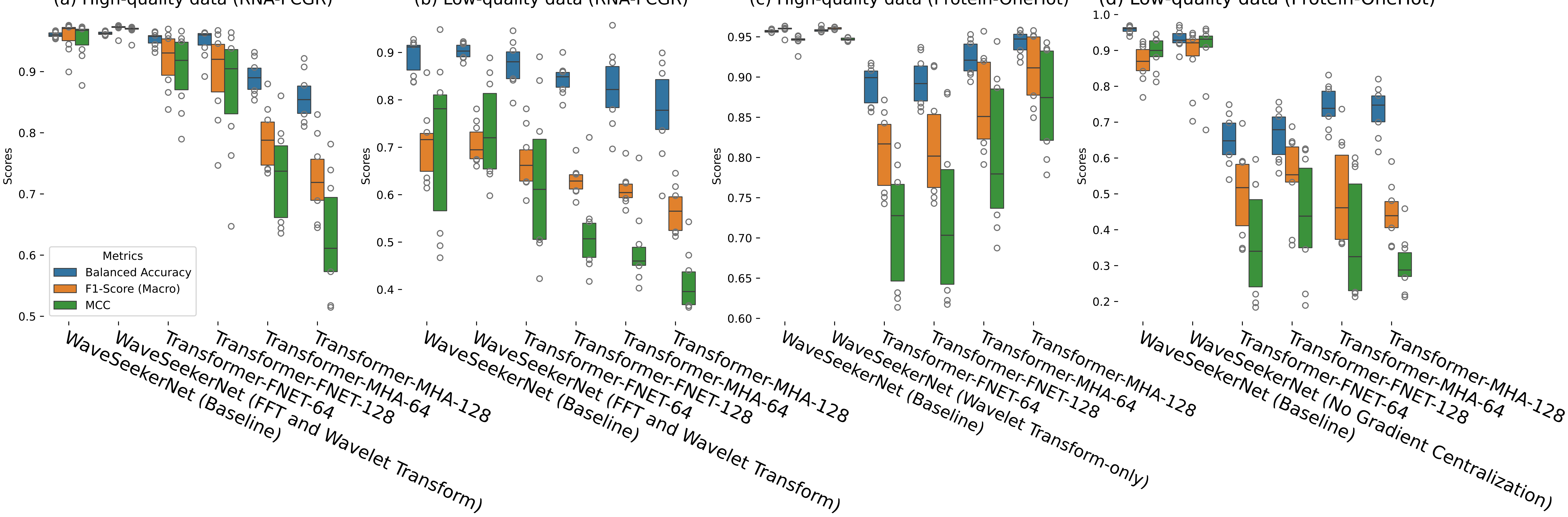

Figure 5

Click here to access/download;Figure;Figure 5.pdf

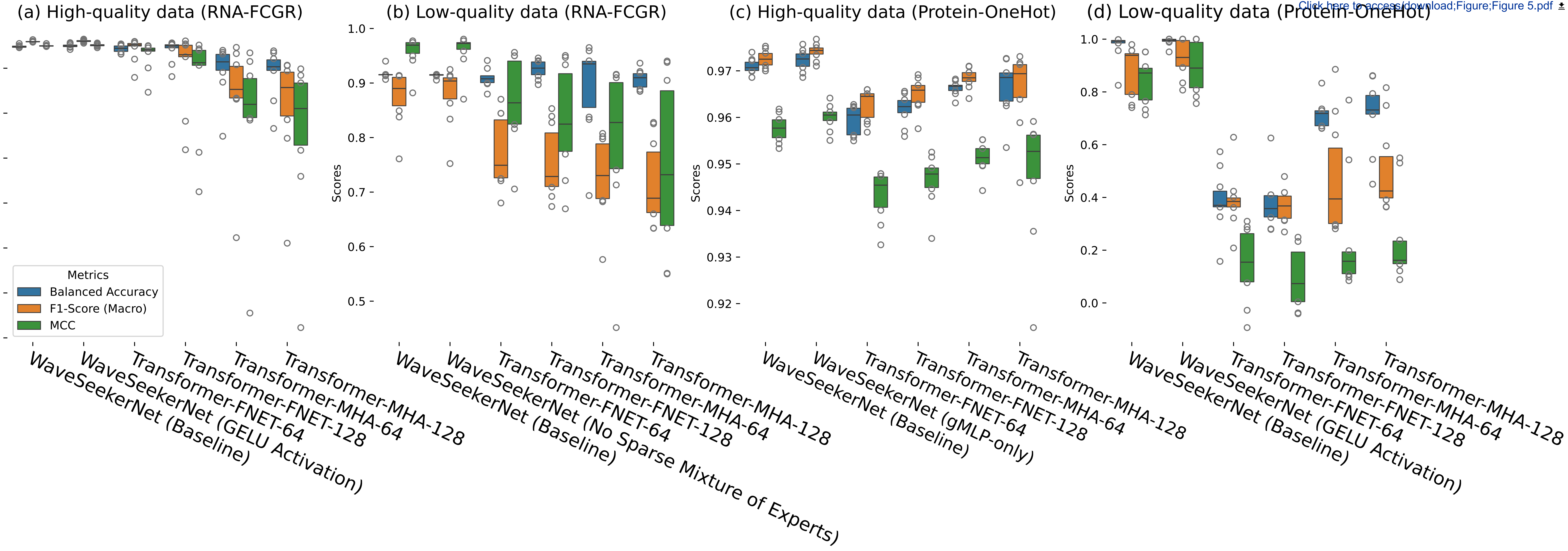

Figure 6

Click here to access/download;Figure;Figure 6.pdf

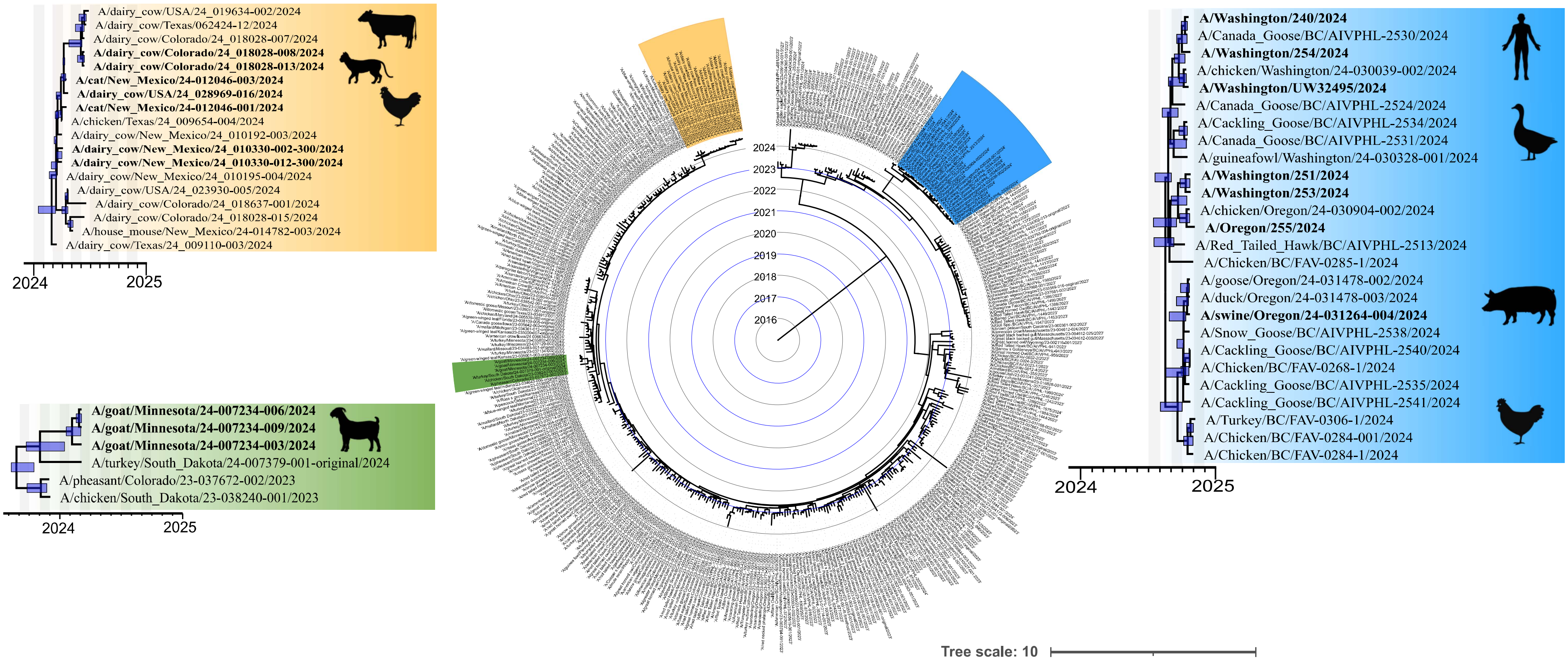

Supplementary Figures

Click here to access/download
Supplementary Material
Supplementary Figures.pdf

Supplementary Tables

Click here to access/download
Supplementary Material
Supplementary Tables.xlsx

Click here to access/download
Supplementary Material
Supplementary Algorithms.pdf

Personal Cover Click here to access/download;Personal Cover;Cover_Letter.pdf

Dr. Scott Edmunds
Editor in Chief, *GigaScience*

February 24, 2025

Dear Dr. Scott Edmunds,

We are writing this email to submit a manuscript for consideration as a Research Article in *GigaScience*. Our study, "*WaveSeekerNet: Accurate Prediction of Influenza A Virus Subtypes and Host Source Using Attention-Based Deep Learning*", presents a novel deep learning model for accurate and rapid prediction of Influenza A Virus (IAV) subtypes and host source.

Influenza A virus is a global health threat that can spread between animals and humans, causing outbreaks and pandemics. Since 1918, four major pandemics have occurred. In December 2021, Highly Pathogenic Avian Influenza H5N1 outbreaks occurred in North America, since then this virus has affected millions of birds in Canada and the United State, spread to mammals, including humans.

Deep learning has emerged as a powerful tool for studying viruses and understanding the factors influencing their spread and evolution. By applying and further developing modern deep-learning algorithms, we have created a unique model called WaveSeekerNet and empirically demonstrated its ability to accurately detect influenza virus subtypes, host sources, and early cross-species transmission events. We highlight these findings and discuss their importance in enhancing IAV surveillance and pandemic preparedness.

We believe that this study provides a valuable resource for combating Influenza A Virus and are confident that this study will attract readers in wide range of fields such as Computer Science, Computational Biology, Virology. Thus, we think that our work is suitable for publication in *GigaScience*.

This manuscript is original research and is not being considered for publication elsewhere. All authors have read and consent to submit this manuscript to *GigaScience* and have no conflicts of interest to disclose.

For your convenience, we suggest following referees: Steven Van Borm (Belgian Institute for Health; Steven.VanBorm@sciensano.be), Ashkan Ebadi (National Research Council Canada – University of Waterloo – Concordia University; Ashkan.Ebadi@nrc-cnrc.gc.ca), Chandana Tennakoon (The Pirbright Institute - chandana.tennakoon@pirbright.ac.uk), Chih-Yu Chen (Public Health Agency of Canada; chih-yu.chen@phac-aspc.gc.ca).

We highly appreciate your consideration and look forward to hearing feedback from you.

Sincerely yours,

Hoang-Hai Nguyen and Josip Rudar (on behalf of all authors)
