# Supplementary material for: WaveSeekerNet: accurate prediction of influenza A virus subtypes and host source using attention-based deep learning
Source: Gigascience. 2025 Aug 29;14:giaf089. doi: 10.1093/gigascience/giaf089 (PMC12395966; doi:10.1093/gigascience/giaf089)
Supplement: giaf089_Authors_Response_To_Reviewer_Comments_Original_Submission [file giaf089_authors_response_to_reviewer_comments_original_submission.pdf]

Author's Response To Reviewer Comments

I. General responses:

Based on the reviewers' comment: "Could the oversampling/undersampling methods employed in the study introduce any potential biases to the analysis?", we realized that different biases can be introduced depending on where resampling occurs. This is supported by the study by Aydin Demircioğlu, "Applying oversampling before cross-validation will lead to high bias in radiomic." In our original submission, we performed upfront resampling on the entire pre-2020 dataset, and the resampled training data will be used for the cross-validation loop (we called Strategy B in this revision); the models will be evaluated by the independent test sets. However, this approach could have led to an overly optimistic model. In this revision, we changed the methods used to train all models. Specifically, the entire pre-2020 training data will be used for repeated stratified 10-fold cross-validation, and resampling will now be performed within the cross-validation loop (we called Strategy A in this revision). Therefore, we re-ran all experiments, performed additional experiments for the pre-trained ESM-2 model, and conducted in-depth analyses. As expected, the results from these re-run experiments did not change our original findings and conclusions. However, the precise statistics/numbers were also updated to reflect the re-run experiments.

Furthermore, we have fixed grammar/errors/typos and updated words and sentences to improve the manuscript's readability and flow. We also fixed other writing errors, such as Equation 1 in Section 2.2, Algorithm S3 and Equation 1 of Algorithm S4 in the Supplementary Algorithm. In addition to specific comments about the figures in the experiments, we also updated Figure 1 to clearly describe our methods of collecting data and training models. Figure 2 was also fixed for errors and updated to give the audience more details and clarity about our models (e.g., Figure 2b (grammar/typos in MH-SMoE layer), 2e (added gMLP block), and 2f (fixed for drawing errors and updated with more details)).

Please find our detailed responses to the comments we received and a description of the changes. We submitted the revised manuscript with tracked changes so the reviewers can see what changes/updates have been made since the original submission.

II. Response to editor:

Editor: In addition, please register any new software application in the bio.tools and SciCrunch.org databases to receive RRID (Research Resource Identification Initiative ID) and biotoolsID identifiers, and include these in your manuscript. Computational workflows should be registered in workflowhub.eu and the DOIs cited in the relevant places in the manuscript. These will facilitate tracking, reproducibility and re-use of your tool.

[Author's response]: Thank you for your feedback. We have registered resources for WaveSeekerNet and included RRID:SCR_026942 and biotools:waveseekernet into the manuscript.

III. Response to reviewers:

Reviewer #1: The manuscript presented by Nguyen et al. is well written, well researched, and well executed. The use of this new "wavelet style" neural network shows both an increased training efficiency and improved accuracy at detecting influenza subtypes for surveillance. However, I think their comparison to a 'plain' Transformer model does not take advantage of the improvements in pre-training and transfer-learning that have become standard practice in deep-learning. I have also included some stylistic suggestions to improve the figures as presented. After addressing these comments, I believe that this will become a very strong manuscript.

Major Comments:

1. The authors present a comparison between their new wavelet architecture and a standard transformer

architecture using a one-hot encoded vector of amino-acids. I believe that this is the correct 'null model' to compare your wavelet architecture to, however, it does not represent the 'state of the art' in utilizing transformers for sequence analysis. As I'm sure the authors are aware, the disadvantage of transformers is that they take an extensive amount of training (they note the transformer only models take 2-4X more training epochs to converge). However, the advantage they bring is that they can be extensively trained for one task and then transfer that learning to another related task. A number of models have been pre-trained on giant collections of proteins Asgari et al, https://doi.org/10.1371/journal.pone.0141287 and Rives et al https://doi.org/10.1073/pnas.2016239118 which then allow one to transfer that knowledge to different domains with fewer examples such as demonstrated in Dampier et al https://doi.org/10.3389/fviro.2022.880618. It would be interesting to see whether your wavelet model defeats these pre-trained models with transfer learning. If you showed that, you could argue that there is no need for the extensive expense of 'foundational models'.

[Author’s response]: Thank you for this comment. To test the efficacy of transfer learning, we performed additional tests using the pretrained ESM-2 models. Unfortunately, we were unable to test this using the more recent ESM-3 since it was too large to run on the computational infrastructure available to us. However, given our results with ESM-2, we strongly believe that the overall picture is unlikely to be substantially different.
Summary: The testing results indicated that the pretrained ESM-2 models demanded significantly more training time than WaveSeekerNet and Transformer-only and only resulted in comparable generalization performance to WaveSeekerNet when tested on the high-quality protein sequences. However, when tested with low-quality datasets, the pre-trained ESM-2 still lagged behind WaveSeekerNet. For example, with HA subtype prediction using the low-quality sequences, the ESM-2_t6 model achieved an F1-score (Macro Average) of 0.473, while WaveSeekerNet with FFT-based head achieved an F1-score (Macro Average) of 0.932. In terms of parameter count and memory demands, the ESM-2_t12 model has 33.5 million parameters, compared to the baseline WaveSeekerNet with 1.2 million. Their respective training times per epoch are 375 and 18.2 seconds. We invite the reviewers to review Table 2 and Supplementary S8, which compare the WaveSeekerNet, pre-trained ESM-2 and the Transformer-only model.

Briefly, our model defeated the pre-trained ESM-2 models using the methods described here with respect to classification performance and compute/memory requirements.

Updates Made: To address the reviewer’s comments, we made updates as follows:

- Updated Section 4.1 (Model Selection, training, and testing), which describes our methods to test the pre-trained ESM-2 model (lines 359-369) and Table 1 which includes the descriptions of the pretrained ESM-2 models
- Added a new Section 5.2 (WaveSeekerNet achieves excellent subtype and host source classification using a parameter-efficient design).
- Updated Section 5.4 (5.2 in the original submission) (WaveSeekerNet can predict Influenza HA and NA subtypes with high accuracy) (lines 569-573)
- Updated Section 5.5 (5.3 in the original submission) (WaveSeekerNet accurately identifies the host source of using influenza A virus consensus sequences) (lines 624-629 and 634-635)

We would like to thank the reviewer for this comment, as it provided additional strong evidence to show that models with fewer parameters can still be used as strong alternatives to larger, more complex, and expensive approaches.

2. The authors discuss that there is a significant imbalance in the training set and they used up-sampling and limiting to balance out the class representation. Since the classes are not equally represented, the model may not be equally able to predict each class. And the high metrics may only be a representation of its ability to predict the popular classes correctly. The authors should include an additional set of figures (supplemental is fine) that show the metrics broken out by Subtype. It would also be interesting to see a graph of the class-size (before up-sampling) vs F1-score (or another metric) on that class. This could provide lower-bounds for how many samples are needed to train the model.

[Author’s response]: We would like to thank the reviewer for this comment. In this work, we reported results using several evaluation metrics, such as the balanced accuracy score, the macro-weighted F1-score, and the Matthews Correlation Coefficient. A full overview of how we approached evaluating predictive performance can be found in Section 4.4 (Evaluation Metrics).

Updates Made: To address the reviewer’s comments, we updated Section 5.4 (5.2 in the original submission) (WaveSeekerNet can predict Influenza HA and NA subtypes with high accuracy). Lines 562-566 and 592-596 demonstrate WaveSeekerNet’s superiority in correctly identifying common and rare subtypes. For example, the H1 (ntrain= 56,593, ntest= 19,599) and H8 (ntrain= 189, ntest= 12) subtypes, each achieved an F1-score of 1.0, contributing to WaveSeekerNet’s overall F1-score (Macro Average) of 1.0. We invite the reviewer to review Supplementary Tables S15 and S16, which break down the F1-score for specific subtypes, including the number of training sequences (class-size before resampling), number of test sequences. These classification reports provide a better picture of how our resampling procedures and class imbalance impact predictive performance. For data distribution of this study, please refer to Supplementary Tables S1-5.

Minor Comments:

1. Figures 3, 4, and 5: These would benefit from a linked y-axis. It is hard to compare across A/B/C/D when the axes have different y-limits.

[Author’s response]: We thank the reviewer for this comment. We changed to use bar plots and updated y-axis limits (i.e., with the same y-limits) to make it clear for comparisons.

Reviewer #2: Nguyen HH and collaborators trained an ensemble-like deep learning model on HA and NA sequences extracted from GISAID (sequences collected from 1902 to 2019) to predict 1/influenza subtype and 2/host source. Their model was tested on HA and NA sequences collected from 2020 to 2025 and showed improved accuracies compared to other deep learning models. The article is of good quality, with well-documented methods and with proper use of a test set that would mimic real case use of the model (the model would be used on future sequences) and the use of a standard metric to assess the accuracy of the model (F1-score, Bal. Acc, MCC). The figures and tables support the conclusions of the article.

I only have two minor edits that I would suggest to the authors:

1. In the first paragraph of the introduction, the authors explain why predicting host sources is important (for active surveillance and our preparedness for future pandemics). Can the authors explain why predicting influenza subtype is also crucial?

[Author’s response]: We thank the reviewer for this comment. We updated the introduction on lines 83-86 to explain why subtype prediction is an important task.

2. lines 573-575. The authors argue that their model is better suited to predict rare variants than previous models like MC-NN. Do the authors think this is only the result of the upsampling of those sequences?

[Author’s response]: We thank the reviewer for this comment. The resampling process was applied only to sequences collected before 2020, which were used to train each model. Furthermore, all models were trained using the same set of resampled training data and subsequent tests were conducted using the independent test sets (see our revised Figure 1 for an overview of the training/evaluation procedures). In the MC-NN paper, Xu et al. considered rare subtypes (e.g., H15, H17, H18, N10 and N11) together as a single group when testing the attention-based MC-NN model and evaluated the performance of MC-NN using a weighted one-vs-all strategy. Consequently, their reported F1-scores are likely biased towards the majority classes, potentially leading to overly optimistic performance assessments. In this instance, we believe this outcome is not attributable to the up-sampling of rare subtypes, but rather to the intrinsic

performance of the MC-NN model itself. You can also refer to Supplementary Tables S15 and S16, which break down the F1-score for specific subtypes, including the number of training sequences (class-size before resampling), number of test sequences. These classification reports provide a better picture of WaveSeeker's and MC-NN's (Transformer-only) performance. From these tables, you can see that MC-NN (Transformer-only) achieved a very high F1-Score (Weighted Avg) but still very low with F1-Score (Macro Avg).

Reviewer #3:
(1) In the abstract, the statement 'WaveSeekerNet achieves scores of up to the maximum 1.0 across all evaluation metrics, including F1-score (Macro Average)' appears to slightly deviate from the actual experimental results.

[Author's response]: We thank the reviewer for this comment. We updated the abstract on lines 34-37 to make it clearer and consistent with actual experimental results on lines 558-560 and 590-592 of Section 5.4 (WaveSeekerNet can predict Influenza HA and NA subtypes with high accuracy).

(2) In data preprocessing, the reasoning behind selecting and keeping the earliest collected sequence when duplicate sequences are encountered should be explained.

[Author's response]: We thank the reviewer for this comment. We added clarifications on lines 128-131 of Section 2.1. When duplicate sequences are found across different hosts, there's no definitive method for selecting which to retain. However, we aim to identify the most likely original host for each sequence. The earliest collected sample provides the best available approximation of which host first contributed that sequence to our dataset. This temporal approach offers a straightforward and defensible criterion that aligns with our goal of tracing sequence origins.

(3) Compared with Figure 4, Figure 5 demonstrates performance improvements in most cases, but why is this not observed for some results in Figure 4d?

[Author's response]: We thank the reviewer for this comment. We updated figures using the bar plots and fixed the y-axis limit for easier comparison.
We wanted to show the comparisons when testing with high-quality datasets (please refer to Section 2.1 and Figure 1, where we described what high-quality, low-quality datasets are). Figures 4a, 4c show the comparison when predicting host source using the HA segment, and WaveSeekerNet is better than Transformer-only models on the high-quality dataset. Supplementary Figures S7a, S7c and Figures 5a, 5c show the comparison when predicting host source using NA segment and the combined HA & NA segments (or 2 channels), respectively. In these tests, the Transformer-only model is comparable to WaveSeekerNet. We also wanted to show the comparisons when testing with low-quality datasets. Here, we show that the Transformer-only models have significantly lower permanence than WaveSeekerNet, especially with protein sequences (Figure 4d and 5d). We believe this outcome is due to the intrinsic performance of the Transformer models, as demonstrated with subtype prediction. To have concrete comparisons, we performed additional tests with the pretrained ESM-2 models using transfer learning (as suggested by Reviewer #1). The ESM-2 model showed improvements compared to Transformer-only models, yet it did not reach the performance of WaveSeekerNet on the low-quality data.

(4) Could the oversampling/undersampling methods employed in the study introduce any potential biases to the analysis?

[Author's response]: We thank the reviewer for this comment. In general, the resampling may indeed introduce biases into an analysis. However, the impact of bias may happen depending on where resampling is applied. In the original submission, we resampled the entire pre-2020 training data before cross-validation. The models were evaluated using the independent post-2020 test sets (which were not resampled). We chose this approach (now is Strategy B in this revision) to maintain the diversity and the same number of classes for the training set in each cross-validation fold. Otherwise, some classes such as H15 (5 protein sequences), H17 (2 sequences), H18 (1 sequence), N10 (2 sequences), and N11 (1

sequence) may only be seen in single fold or several folds. As mentioned in the General responses, we changed methods to train the models, Strategy A, in this revision. We re-ran experiments and updated the results for the manuscript.

Updates Made: To address the reviewer's comments, we made updates as follows:
- Updated Section 4.1 (Model Selection, training, and testing) and Figure 1, which describes resampling approaches.
- Updated results through the text with re-run experiments.
- Added new Section 5.3 (WaveSeekerNet is robust to different up-sampling and down-sampling strategies). We performed in-depth analyses using the Bayesian correlated t-test and Trinomial test to statistically compare WaveSeekerNet's performance under two resampling strategies (Strategy A vs Strategy B). Our results showed that the choice of resampling approach has little impact on the ability of WaveSeekerNet to generalize in both the subtype prediction and host source prediction tasks. For example, when predicting host source using the high-quality RNA sequences of the HA segment, the baseline model achieved an F1-score (Macro Average) of 0.969±0.008 if up-sampling and down-sampling were performed before cross-validation, versus 0.965±0.015 if they were only performed on each training split. These two scenarios had a 100% probability of being within the ROPE, indicating that the difference between the two resampling methods is negligible. We further evaluated if there was any bias by using the Trinomial test; we showed that the difference in WaveSeekerNet's performance between the two resampling approaches is negligible, which demonstrates the robustness of WaveSeekerNet to different resampling strategies. We invite the reviewers to review the Supplementary Table S9-14, which shows the results of in-depth analyses.

We would like to thank the reviewer for this comment, which helped us improve our method and further emphasized our approach's strengths.

(5) Given that VADR can provide viral classification and annotation information-which serves as the benchmark in this study, what specific advantages does WaveSeekerNet offer for subtype classification?

[Author's response]: We thank the reviewer for this comment. Our comparison to VADR primarily serves as a sanity check on our model. Since our model outperformed MC-NN and pre-trained models, such as ESM-2, this demonstrated competitive performance against established baselines. However, in a practical sense, our improvement over these approaches would not be meaningful if our model performed poorly when compared to the gold standard. Since WaveSeekerNet is competitive with VADR, it can serve as an additional source of evidence to support diagnostic results produced with more traditional methods and improve the robustness of these analyses, given that WaveSeekerNet does not rely as heavily on expert annotations. We updated the discussion section to discuss additional advantages of WaveSeekerNet on lines 768-781

(6) The paper employs 10-fold cross-validation to assess generalizability, yet the data processing section describes a temporal split (pre-2020 for training). Could the "Model Training and Testing" section provide further clarification on this?

[Author's response]: We thank the reviewer for this comment. We expanded Section 4.1 (Model Selection, Training and Testing) and updated Figure 1 for more details. The updates also included the methods for the pre-trained ESM-2 models (as suggested by Reviewer #1) and the details on resampling strategies.

Cookie Preference Center

We use cookies which are necessary to make our site work. We may also use additional cookies to analyze, improve and personalize our content and your digital experience. For more information, see our Cookie Policy.

You may choose not to allow some types of cookies. However, blocking some types may impact your experience of our site and the services we are able to offer. See the different category headings below to find out more or change your settings.

Allow all

Manage Consent Preferences

Strictly Necessary Cookies

Always active

These cookies are necessary for the website to function and cannot be switched off in our systems. They are usually only set in response to actions made by you which amount to a request for services, such as setting your privacy preferences, logging in or filling in forms. You can set your browser to block or alert you about these cookies, but some parts of the site will not then work.

Functional Cookies

These cookies enable the website to provide enhanced functionality and personalisation. They may be set by us or by third party providers whose services we have added to our pages. If you do not allow these cookies then some or all of these services may not function properly.

Performance Cookies

These cookies allow us to count visits and traffic sources so we can measure and improve the performance of our site. They help us to know which pages are the most and least popular and see how visitors move around the site.

Targeting Cookies

These cookies may be set through our site by our advertising partners. They may be used by those companies to build a profile of your interests and show you relevant adverts on other sites. If you do not allow these cookies, you will experience less targeted advertising.

Cookie List

Clear

Apply Cancel
Consent Leg.Interest

Confirm my choices

Powered by onetrust
