# Supplementary material for: WaveSeekerNet: accurate prediction of influenza A virus subtypes and host source using attention-based deep learning
Source: Gigascience. 2025 Aug 29;14:giaf089. doi: 10.1093/gigascience/giaf089 (PMC12395966; doi:10.1093/gigascience/giaf089)
Supplement: giaf089_Reviewer_1_Report_Original_Submission — Will Dampier -- 3/18/2025 [file giaf089_reviewer_1_report_original_submission.pdf]

Reviewer Report

Title: WaveSeekerNet: Accurate Prediction of Influenza A Virus Subtypes and Host Source Using Attention-Based Deep Learning

Version: Original Submission **Date:** 3/18/2025

Reviewer name: Will Dampier

Reviewer Comments to Author:

The manuscript presented by Nguyen et al. is well written, well researched, and well executed. The use of this new "wavelet style" neural network shows both an increased training efficiency and improved accuracy at detecting influenza subtypes for surveillance. However, I think their comparison to a 'plain' Transformer model does not take advantage of the improvements in pre-training and transfer-learning that have become standard practice in deep-learning. I have also included some stylistic suggestions to improve the figures as presented. After addressing these comments, I believe that this will become a very strong manuscript.Major Comments:The authors present a comparison between their new wavelet architecture and a standard transformer architecture using a one-hot encoded vector of amino-acids. I believe that this is the correct 'null model' to compare your wavelet architecture to, however, it does not represent the 'state of the art' in utilizing transformers for sequence analysis. As I'm sure the authors are aware, the disadvantage of transformers is that they take an extensive amount of training (they note the transformer only models take 2-4X more training epochs to converge). However, the advantage they bring is that they can be extensively trained for one task and then transfer that learning to another related task. A number of models have been pre-trained on giant collections of proteins Asgari et al, https://doi.org/10.1371/journal.pone.0141287 and Rives et al https://doi.org/10.1073/pnas.2016239118 which then allow one to transfer that knowledge to different domains with fewer examples such as demonstrated in Dampier et al https://doi.org/10.3389/fviro.2022.880618. It would be interesting to see whether your wavelet model defeats these pre-trained models with transfer learning. If you showed that, you could argue that there is no need for the extensive expense of 'foundational models'.The authors discuss that there is a significant imbalance in the training set and they used up-sampling and limiting to balance out the class representation. Since the classes are not equally represented, the model may not be equally able to predict each class. And the high metrics may only be a representation of its ability to predict the popular classes correctly. The authors should include an additional set of figures (supplemental is fine) that show the metrics broken out by Subtype. It would also be interesting to see a graph of the class-size (before up-sampling) vs F1-score (or another metric) on that class. This could provide lower-bounds for how many samples are needed to train the model.Minor Comments:Figures 3, 4, and 5: These would benefit from a linked y-axis. It is hard to compare across A/B/C/D when the axes have different y-limits.

Level of Interest

Please indicate how interesting you found the manuscript: Choose an item.

Quality of Written English

Please indicate the quality of language in the manuscript: Choose an item.

Declaration of Competing Interests

Please complete a declaration of competing interests, considering the following questions:

- Have you in the past five years received reimbursements, fees, funding, or salary from an organisation that may in any way gain or lose financially from the publication of this manuscript, either now or in the future?
- Do you hold any stocks or shares in an organisation that may in any way gain or lose financially from the publication of this manuscript, either now or in the future?
- Do you hold or are you currently applying for any patents relating to the content of the manuscript?
- Have you received reimbursements, fees, funding, or salary from an organization that holds or has applied for patents relating to the content of the manuscript?
- Do you have any other financial competing interests?
- Do you have any non-financial competing interests in relation to this paper?

If you can answer no to all of the above, write 'I declare that I have no competing interests' below. If your reply is yes to any, please give details below.

I declare that I have no competing interests

I agree to the open peer review policy of the journal. I understand that my name will be included on my report to the authors and, if the manuscript is accepted for publication, my named report including any attachments I upload will be posted on the website along with the authors' responses. I agree for my report to be made available under an Open Access Creative Commons CC-BY license (http://creativecommons.org/licenses/by/4.0/). I understand that any comments which I do not wish to be included in my named report can be included as confidential comments to the editors, which will not be published.

Choose an item.

To further support our reviewers, we have joined with Publons, where you can gain additional credit to further highlight your hard work (see: https://publons.com/journal/530/gigascience). On publication of this paper, your review will be automatically added to Publons, you can then choose whether or not to claim your Publons credit. I understand this statement.

Yes Choose an item.
