# Supplementary material for: WaveSeekerNet: accurate prediction of influenza A virus subtypes and host source using attention-based deep learning
Source: Gigascience. 2025 Aug 29;14:giaf089. doi: 10.1093/gigascience/giaf089 (PMC12395966; doi:10.1093/gigascience/giaf089)
Supplement: giaf089_Reviewer_2_Report_Original_Submission — Slim Fourati -- 3/31/2025 [file giaf089_reviewer_2_report_original_submission.pdf]

Reviewer Report

Title: WaveSeekerNet: Accurate Prediction of Influenza A Virus Subtypes and Host Source Using Attention-Based Deep Learning

Version: Original Submission **Date:** 3/31/2025

Reviewer name: Slim Fourati

Reviewer Comments to Author:

Nguyen HH and collaborators trained an ensemble-like deep learning model on HA and NA sequences extracted from GISAID (sequences collected from 1902 to 2019) to predict 1/influenza subtype and 2/host source. Their model was tested on HA and NA sequences collected from 2020 to 2025 and showed improved accuracies compared to other deep learning models. The article is of good quality, with well-documented methods and with proper use of a test set that would mimic real case use of the model (the model would be used on future sequences) and the use of a standard metric to assess the accuracy of the model (F1-score, Bal. Acc, MCC). The figures and tables support the conclusions of the article.I only have two minor edits that I would suggest to the authors:1. In the first paragraph of the introduction, the authors explain why predicting host sources is important (for active surveillance and our preparedness for future pandemics). Can the authors explain why predicting influenza subtype is also crucial?2. lines 573-575. The authors argue that their model is better suited to predict rare variants than previous models like MC-NN. Do the authors think this is only the result of the upsampling of those sequences?

Level of Interest

Please indicate how interesting you found the manuscript: Choose an item.

Quality of Written English

Please indicate the quality of language in the manuscript: Choose an item.

Declaration of Competing Interests

Please complete a declaration of competing interests, considering the following questions:

- Have you in the past five years received reimbursements, fees, funding, or salary from an organisation that may in any way gain or lose financially from the publication of this manuscript, either now or in the future?
- Do you hold any stocks or shares in an organisation that may in any way gain or lose financially from the publication of this manuscript, either now or in the future?
- Do you hold or are you currently applying for any patents relating to the content of the manuscript?

- Have you received reimbursements, fees, funding, or salary from an organization that holds or has applied for patents relating to the content of the manuscript?
- Do you have any other financial competing interests?
- Do you have any non-financial competing interests in relation to this paper?

If you can answer no to all of the above, write 'I declare that I have no competing interests' below. If your reply is yes to any, please give details below.

I declare that I have no competing interests

I agree to the open peer review policy of the journal. I understand that my name will be included on my report to the authors and, if the manuscript is accepted for publication, my named report including any attachments I upload will be posted on the website along with the authors' responses. I agree for my report to be made available under an Open Access Creative Commons CC-BY license (http://creativecommons.org/licenses/by/4.0/). I understand that any comments which I do not wish to be included in my named report can be included as confidential comments to the editors, which will not be published.

Choose an item.

To further support our reviewers, we have joined with Publons, where you can gain additional credit to further highlight your hard work (see: https://publons.com/journal/530/gigascience). On publication of this paper, your review will be automatically added to Publons, you can then choose whether or not to claim your Publons credit. I understand this statement.

Yes Choose an item.
